# The Wor1-like Protein Fgp1 Regulates Pathogenicity, Toxin Synthesis and Reproduction in the Phytopathogenic Fungus *Fusarium* g*raminearum*

**DOI:** 10.1371/journal.ppat.1002724

**Published:** 2012-05-31

**Authors:** Wilfried Jonkers, Yanhong Dong, Karen Broz, H. Corby Kistler

**Affiliations:** 1 Department of Plant Pathology, University of Minnesota, St. Paul, Minnesota, United States of America; 2 USDA-ARS, Cereal Disease Laboratory, St. Paul, Minnesota, United States of America; University of Melbourne, Australia

## Abstract

*WOR1* is a gene for a conserved fungal regulatory protein controlling the dimorphic switch and pathogenicity determents in *Candida albicans* and its ortholog in the plant pathogen *Fusarium oxysporum*, called *SGE1*, is required for pathogenicity and expression of key plant effector proteins. *F. graminearum*, an important pathogen of cereals, is not known to employ switching and no effector proteins from *F. graminearum* have been found to date that are required for infection. In this study, the potential role of the *WOR1*-like gene in pathogenesis was tested in this toxigenic fungus. Deletion of the *WOR1* ortholog (called *FGP1*) in *F. graminearum* results in greatly reduced pathogenicity and loss of trichothecene toxin accumulation in infected wheat plants and *in vitro*. The loss of toxin accumulation alone may be sufficient to explain the loss of pathogenicity to wheat. Under toxin-inducing conditions, expression of genes for trichothecene biosynthesis and many other genes are not detected or detected at lower levels in Δ*fgp1* strains. *FGP1* is also involved in the developmental processes of conidium formation and sexual reproduction and modulates a morphological change that accompanies mycotoxin production *in vitro*. The Wor1-like proteins in *Fusarium* species have highly conserved N-terminal regions and remarkably divergent C-termini. Interchanging the N- and C- terminal portions of proteins from *F. oxysporum* and *F. graminearum* resulted in partial to complete loss of function. Wor1-like proteins are conserved but have evolved to regulate pathogenicity in a range of fungi, likely by adaptations to the C-terminal portion of the protein.

## Author Summary

Plant pathogenic fungi can have devastating effects on crop yield and quality. In addition, these fungi may generate mycotoxins that pose health risks when the contaminated crops are consumed. The pathogen *Fusarium graminearum* infects wheat heads and grows through the rachis by synthesizing trichothecene toxins. The mechanisms and environmental cues triggering the production of trichothecene toxins have been studied for many years. Here, we describe a fungal gene, *Fgp1*, that is absolutely required for pathogenicity and mycotoxin synthesis during infection and in culture. *Fgp1* is not required for vegetative growth of *F. graminearum* but is important for reproductive development and potentially for a putative switch from a vegetative to a pathogenic phase. Deletion of *Fgp1* results in reduced expression of the trichothecene biosynthetic gene cluster and genes specifically expressed during toxin synthesis *in planta* and *in vitro*. Fgp1 contains a conserved N-terminal domain and a divergent in the C-terminal region. The corresponding C-terminus from a sister species, *F. oxysporum*, is unable to function fully in *F. graminearum* when fused to the conserved *F. graminearum* N-terminal domain, suggesting the gene function is highly species specific.

## Introduction

Pathogenic fungi have evolved sophisticated ways to infect their hosts, mainly by adapting to the host environment and by producing pathogenicity-related products such as toxic secondary metabolites, effector proteins and/or extracellular enzymes. The expression of genes involved in the adaptation to a host and synthesis of pathogenicity factors are under tight regulation to assure successful infection and survival. The pathogenic fungus *Fusarium graminearum* is a devastating pathogen of wheat and barley [1], [2] and modulates its pathogenicity largely by regulating a cluster of genes encoding enzymes for the biosynthesis of trichothecene toxins [3], [4], [5]. These mycotoxins are required during wheat infection to breach the rachis node of spikelets which acts as a barrier to systemic infection and maximal head blight symptoms. Toxin production during infection depends on multiple cellular and environmental factors (see recent review [6]) and yet, exactly how the genes for trichothecene biosynthesis are regulated is still largely unknown.

Another member of the *Fusarium* genus, *F. oxysporum* [7], contains both pathogenic and non-pathogenic strains. Pathogenic *F. oxysporum* strains modulate their pathogenicity in part by secreting small secreted proteins which may act both as virulence and as avirulence factors [8]. In the tomato wilt pathogen *F. oxysporum* f. sp. *lycopersici*, the nuclear protein Sge1 was demonstrated to be required for parasitic growth and expression of the small-secreted proteins genes *SIX1*, *SIX2*, *SIX3* and *SIX5*, during conditions mimicking *in planta* growth [9]. A Δ*sge1* mutant was still able to colonize roots, but was unable to reach the normally infected xylem vessels. Recently an orthologous gene in the necrotrophic plant pathogen *Botrytis cinerea*, *REG1*, was shown to be required for infection of bean leaves [10], indicating that these genes may have a conserved role in fungal plant pathogenicity.

*SGE1* and *REG1* are orthologs of *WOR1* from *Candida albicans* and *RYP1* in *Histoplasma capsulatum* [11], [12]. In these human pathogenic fungi, both proteins are involved in the dimorphic switch, a transition correlated with the ability to cause disease [13], [14]. This suggests that the orthologous proteins found in plant pathogens also may be involved in switching from a saprophytic to a parasitic lifestyle. In this study we characterize the ortholog of *WOR1* in *F. graminearum*, and show that it is absolutely required for pathogenicity and regulates the expression of the trichothecene biosynthetic (*TRI*) genes *in planta* as well as *in vitro*. It also apparently mediates a morphological change during toxin production, and, as determined by transcriptome analysis, regulates the expression of other genes, many of which are related to pathogenicity.

In order to study functional conservation of the orthologs from the two *Fusarium* species, we interchanged the genes and as well as swapped the C-terminal portions of the genes between the species. We found that the gene from the opposite species was not fully functional in the other species, nor were the chimeric genes. Additionally, very little overlap was found among genes regulated by each protein during vegetative growth. The results from these experiments show that the family of Wor1-like proteins regulates pathogenicity in many fungi through transcriptional reprogramming. Additionally, two members in two closely related *Fusarium* species have evolved different function, presumably in order to adapt to successfully infect their different hosts.

## Results

### Conservation of WOR1-like genes in *Fusarium*

Most fungi have two homologous proteins encoded by the *WOR1* gene family [9], which are recognized by a common *GTI1*/*PAC2* domain, named after the *WOR1*-like genes *GTI1* and *PAC2* of *Schizosaccharomyces pombe* [15], [16]. Phylogenetically, the two paralogous genes from each species sort into two different clades [9] with e.g. *GTI1*, *WOR1*, *RYP1* and *SGE1* residing in one clade [9] and e.g. *PAC2* from *S. pombe* and *PAC2* from *F. oxysporum* in the other clade [9]. In *F. oxysporum SGE1* is required for pathogenicity whereas *PAC2* is not [9]. *F. graminearum* also has these two *WOR1*-like homologs. The gene that shows the highest similarity to *SGE1* from *F. oxysporum* and aligns with the *WOR1* clade is FGSG_12164 that we have named *FGP1* (*F. graminearum GTI1*/*PAC2* 1). The predicted *FGP1* gene is 1029 bp, intronless and encodes a 342 amino acid protein. The paralogous gene from *F. graminearum* that shows the highest similarity to *PAC2* from *F. oxysporum* and aligns with the *PAC2* clade is FGSG_10796 that we have renamed *FGP2* (*F. graminearum GTI1*/*PAC2* 2). The predicted *FGP2* gene is 1266 bp, intronless and encodes a 421 amino acid protein.

In order to assess the conservation of the Fgp1/Sge1 and Fgp2/Pac2 proteins in more *Fusarium* species, we obtained the sequences of the respective proteins from two other sequenced *Fusarium* strains, *F. verticillioides* and *Fusarium solani* f. sp. *pisi* (also known as *Nectria haematococca*), using the BLAST function [17] on the websites of the Broad Institute and the DOE Joint Genome Institute, respectively. As for *F. oxysporum* and *F. graminearum*, two genes were found for *F. verticillioides* as well as for *F. solani*. The genes that show the highest similarity to *SGE1* and *FGP1* and align with the *WOR1* clade are FVEG_09150 and Fs_81912 of *F. verticillioides* and *F. solani*, respectively. The genes that show the highest similarity to *PAC2* and *FGP2* and align with the *PAC2* clade are FVEG_11476 and Fs_60837 of *F. verticillioides* and *F. solani*, respectively.

**Figure 1:**
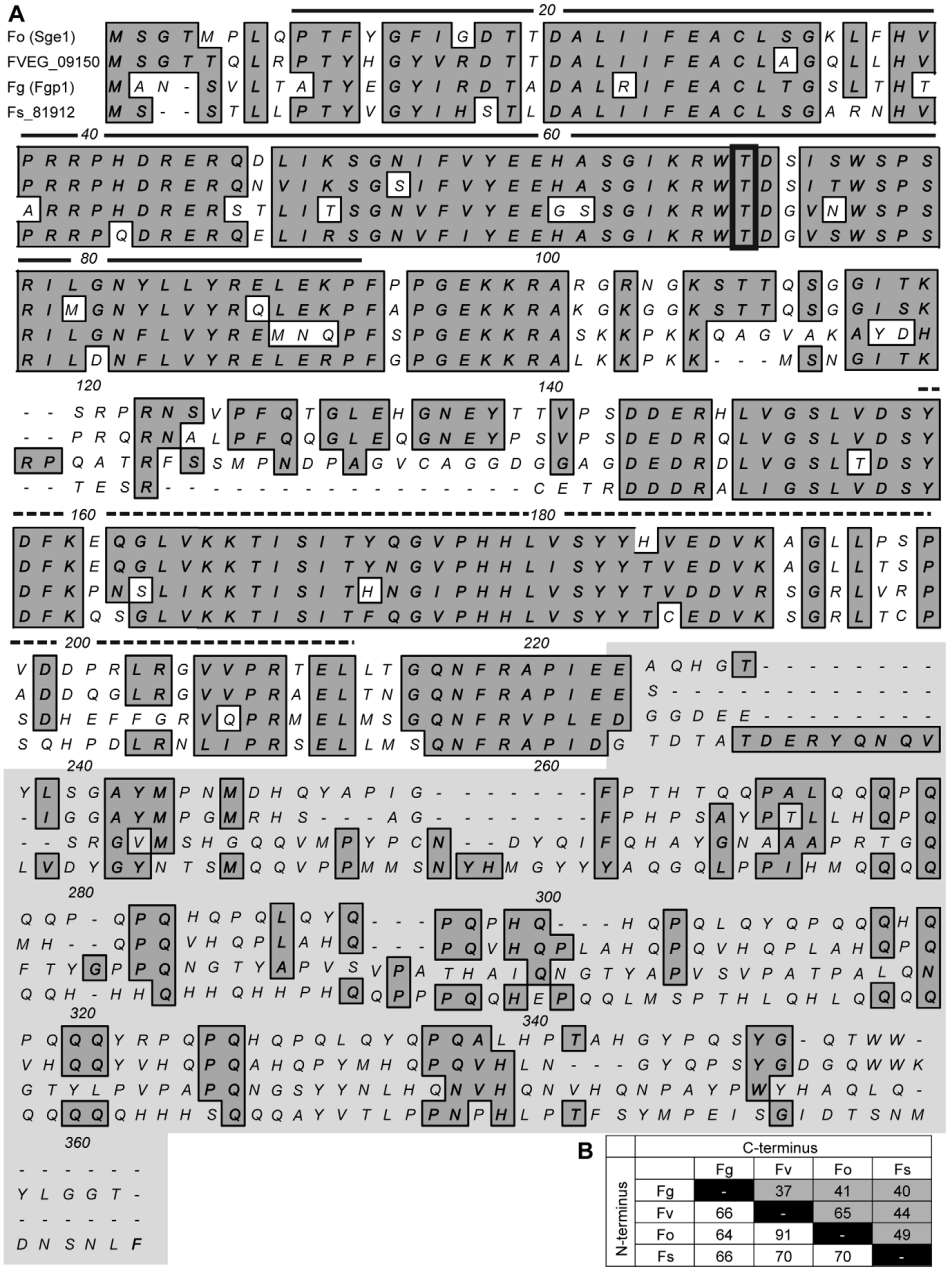
Alignment of four Wor1-like *Fusarium* orthologs. A) Protein sequence alignment of four *Fusarium* Wor1-like proteins: Fo: Sge1 (FOXG_10510) from *F. oxysporum*, FVEG_09150 from *F. verticillioides*, Fg: Fgp1 (FGSG_12164) from *F. graminearum* and Fs: Fs_81912 from *F. solani* (*Nectria haematococca*). Conserved and similar residues are shaded grey. The boxed threonine residue at position 68 is a conserved putative phosphorylation site. The solid black line represents the WOPRa box and the dashed black line the WOPRb box. The C-terminal part is indicated by grey background. The protein alignment was created using MacVector version 10.6.0. B) Table in which the percentages of similarities are given for each of the Wor1-like ortholog (Fg: Fgp1 (FGSG_12164) from *F. graminearum*, Fv: FVEG_09150 from *F. verticillioides*, Fo: Sge1 (FOXG_10510) from *F. oxysporum* and Fs: Fs_81912 from *F. solani* (*Nectria haematococca*)) to each of the other protein in the alignment presented in A. Different percentages are presented for the N-terminal portion (white cells) and the C-terminal portion (grey cells).

When we aligned the four protein sequences of the Wor1 clade (Fo Sge1, FVEG_09150, Fg Fgp1 and Fs_81912), we found great divergence between the sequences of the four *Fusarium* proteins. Conservation is mainly restricted to the N-terminal portion (first ±220 amino acids in Figure 1A) of which the total similarity percentage ranges from 64–90.5% (Figure 1B). The C-terminal portions (last first ±140 amino acids, grey shaded in Figure 1A) of the *Fusarium* orthologs from the Wor1-clade are highly diverged despite of the high numbers of glutamine residues in all four sequences (5.6–13.6%, Table S1B). Overall sequence similarity is as low as 37% between *F. graminearum* and *F. verticillioides*. The sequences of the *F. verticillioides* ortholog and *F. oxysporum* Sge1 share the highest similarity; 65% (Figure 1B). On the contrary, when we aligned the four protein sequences of the Pac2 clade (Fo Pac2, FVEG_11476, Fg Fgp2 and Fs_60837), high similarities and conservation among the four *Fusarium* proteins (80–97%, Table S1A) are observed throughout the N-terminal and C-terminal regions (Figure S1).

The N-terminal portion of all the proteins from the four *Fusarium* strains in both clades contains the conserved WOPRa box (black line above the sequence, Figure 1 and S1) and WOPRb box (dashed black line above the sequence, Figure 1 and S1) [18]. The WOPRa and WOPRb boxes, previously recognized as the *GTI1/PAC2* domain, have been shown to be involved in DNA binding of Wor1 in *C. albicans*. In the WOPRa box, a conserved threonine residue is present (boxed, Figure 1) that functions as a putative phosphorylation site. Mutation of this site in Gti1, Wor1 and Sge1 impairs the function of the respective proteins [9], [15], [19]. It has been shown previously that the N-terminal portion containing the WOPRa and WOPRb boxes from Fo Sge1 and Fo Pac2 align with the same domains from other proteins of the Wor1 family [18] and the same likely holds for the other *Fusarium* species because of the high conservation of these domains among the *Fusarium* proteins. The C-terminal portions of the Wor1-like proteins from *Fusarium* however have not only greatly diverged from each other but also from other fungal species. For example, the C-termini of the *Fusarium* proteins do not align at all with the C-terminus of Wor1 or Reg1 due to differences both in sequence and in length (data not shown).

### FGP1 is required for pathogenicity and toxin production but FGP2 is not

Strains deleted for *FGP1* or *FGP2* were generated in *F. graminearum* using constructs produced in plasmid pPK2HPH-GFP [20] containing a cassette for a hygromycin B resistance gene fused at the C-terminus to GFP and regulated by a *GPD1* promoter and *TRPC* terminator. In the respective plasmids, the cassette is flanked by the up- and downstream sequences (+/−2000 bp) of *FGP1* or *FGP2*. Using *Agrobacterium tumefaciens* mediated transformation, multiple independent transformants were obtained of which five were selected for both *FGP1* or *FGP2*. For each gene, all five proved by PCR and Southern blot to be homologous gene deletions, indicating that no ectopic transformants were obtained (Figure S2).

**Figure 2:**
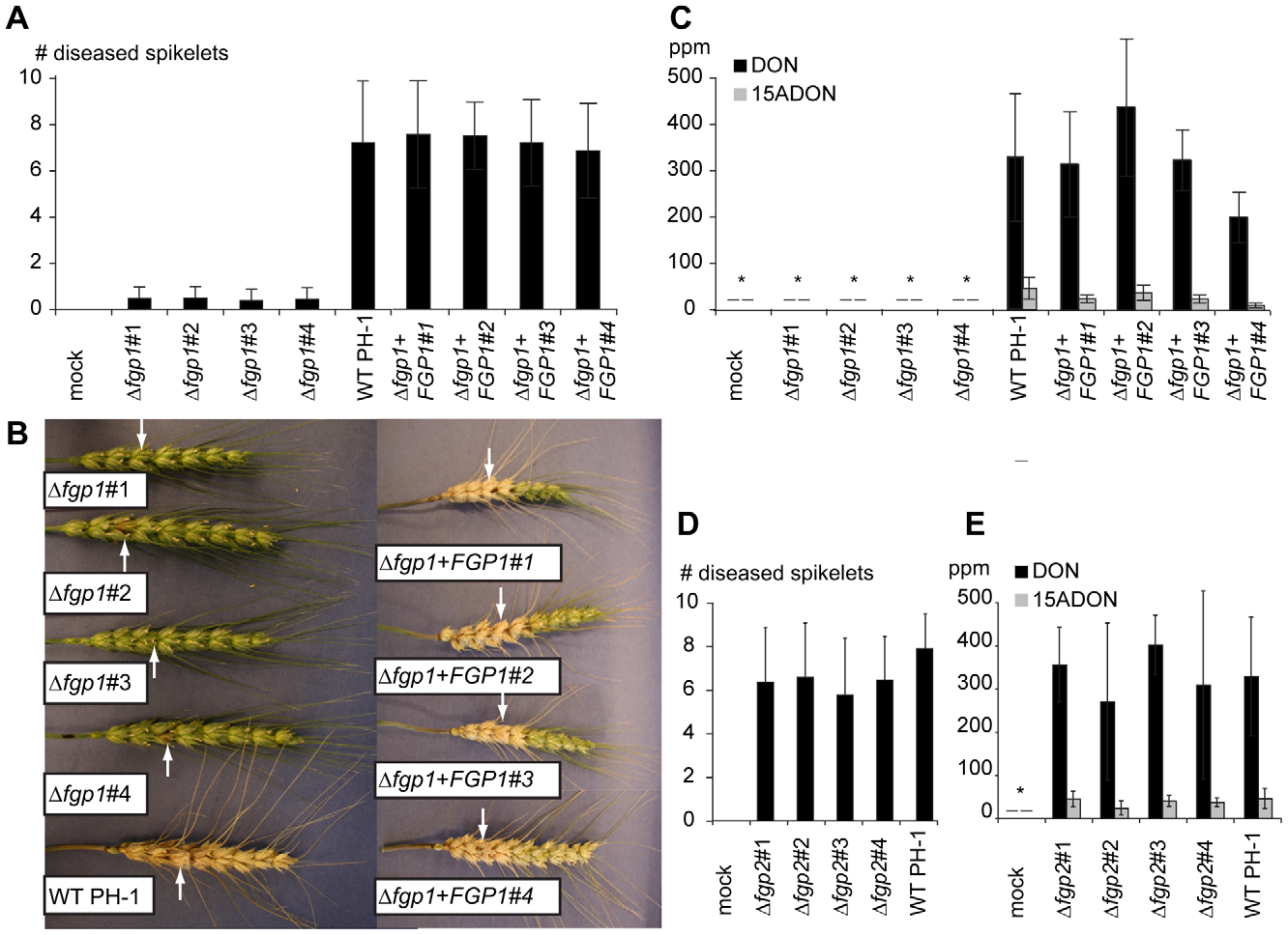
*FGP1* is required for pathogenicity and trichothecene accumulation but *FGP2* is not. A) Average number of diseased spikelets of 30 plants after mock inoculated with H_2_O, inoculated with four independent *FGP1* deletion mutants, with wild type PH-1 or with four independent *FGP1* complemented transformants. Wheat heads were point inoculated and disease spread through adjacent spikelets was enumerated after 14 days. Error bars indicate standard deviation. B) Photographs from wild type inoculated wheat head (lower head on the left), wheat heads inoculated with the four *FGP1* deletion mutants (upper four heads on the left) and wheat heads inoculated with the four *FGP1* complemented transformants (four heads on the right). Arrows indicate the inoculated spikelet in each head photographed. C) DON and 15-ADON concentrations in the inoculated spikelet 14 days after inoculation with H_2_O, four independent *FGP1* deletion mutants, wild type PH-1 or four *FGP1* complemented transformants. Asterisks mean no toxin was detected. D) Average number of diseased spikelets of 30 plants after mock inoculated with H_2_O or inoculated with four independent *FGP2* deletion mutants or wild type PH-1. Wheat heads were point inoculated and spread through adjacent spikelets was enumerated after 14 days. Error bars indicate standard deviation. E) DON and 15-ADON concentrations in the inoculated spikelet 14 days after inoculation with H_2_O, four independent *FGP2* deletion mutants or wild type PH-1. Asterisk means no toxin was detected.

Wheat heads of the variety “Norm”, point-inoculated with the wild type (WT) strain PH-1 and four Δ*fgp1* strains were assessed after two weeks for the number of diseased spikelets. Wild type strain PH-1 was able to spread from the inoculated spikelet (arrow) to other spikelets in the head causing blight symptoms; bleached spikelets and deformed anthers (Figure 2B, left panel, lower head). In contrast, the Δ*fgp1* strains did not spread from the inoculated spikelet (arrows) to other spikelets (Figure 2A). In the inoculated spikelet, the Δ*fgp1* strains only cause minor disease symptoms; some browning of the palea (Figure 2B, left panel, upper four heads). When the inoculated spikelets were analyzed for trichothecene content, no toxin was detected in the spikelets inoculated with the Δ*fgp1* strains, in contrast to spikelets inoculated with the wild type, which accumulated significant levels of the toxins deoxynivalenol (DON) and 15-deoxynivalenol (15-ADON) (Figure 2C).

Reintroduction of the wild type gene *FGP1* into a Δ*fgp1* strain resulted in complemented strains that were tested for spread through the wheat head and for toxin production in the inoculated spikelet. In total, four independent complemented strains used regained the ability to spread throughout the wheat head (Figure 2A) and cause disease symptoms similar to wild type; spread was present from the inoculated spikelets (arrows) to other spikelets in the head (Figure 2B, right panel). The four complemented strains also regained the ability to produce toxin similar to wild type (Figure 2C).

In contrast, Δ*fgp2* strains were not reduced in the ability to cause disease. The Δ*fgp2* strains were able to cause head blight symptoms almost to the same extent as wild type PH-1 (Figure 2D) and accumulated mycotoxin levels comparable to wild type (Figure 2E). These results affirm previous observations that genes from the *WOR1* clade are involved in pathogenicity whereas genes from the *PAC2* clade are not and suggest that *WOR1* orthologs may regulate secondary metabolite synthesis.

### Spread of Δ*fgp1* strains is restricted to the inoculated spikelet

**Figure 3:**
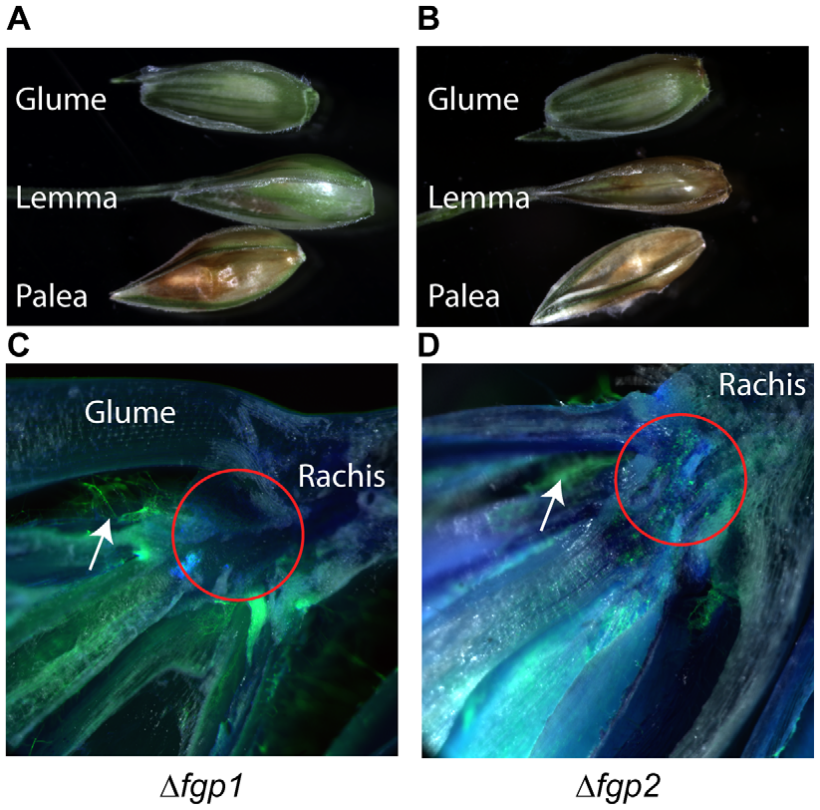
The Δ*fgp1* strain is unable to pass through the rachis node. The infection behavior of the *FGP1* and *FGP2* deletion mutants in the wheat head was determined by light and fluorescence microscopy. Spikelets were inoculated and assessed after two to three days for fungal spread within the floral tissue. A) The palea and lemma of a flower inoculated with Δ*fgp1* show browning. The glume remains green, as no fungal colonization occurs within this portion of the flower. B) The palea, lemma and glume of a flower inoculated with Δ*fgp2* all show browning. C) The GFP expressing Δ*fgp1* strain grows inside the flower but does not penetrate the rachis node. Patches of GFP-expressing fungal mycelium are observed along palea and lemma inside the flower (arrow) but no GFP is seen in the rachis node (red circle) or beyond into the rachis. D) The GFP expressing virulent Δ*fgp2* strain grows in the flower and penetrates the rachis node. Patches of GFP expressing fungal mycelium are observed along palea and lemma inside the flower and GFP is seen within the rachis node (red circle), the rachis and beyond.

As previously mentioned, no spread of disease symptoms was observed with the Δ*fgp1* mutant compared to wild type. To determine the reason for this, we investigated whether the symptoms in the Δ*fgp1* and Δ*fgp2* mutants are correlated to the growth of the mutant strains within the wheat head, using bright light and fluorescent microscopy. Since no differences in symptoms were observed between wild type PH-1 and Δ*fgp2*, the latter was used as a fluorescent positive control strain. After inoculation, spread of the Δ*fgp1* and Δ*fgp2* strains were monitored through the spikelet for two weeks. At three days after inoculation, “fluffy” mycelium growing from of the inoculated spikelet was observed for the Δ*fgp1* strain to the same extent as wild type (data not shown) and the Δ*fgp1* strain had infected the palea and a portion of the lemma but had not spread towards the glume on the outer part of the spikelet (Figure 3A). Even after two weeks, the glume was still green and apparently healthy. Presence of the fungus in the palea and lemma is manifested by browning of the tissue and was verified by microscopy (data not shown). In contrast to Δ*fgp1*, the Δ*fgp2* strain had infected the palea, the lemma and had spread to the glume after three days (Figure 3B). For both Δ*fgp1* and Δ*fgp2* strains, *GFP* is coupled to the constitutively expressed hygromycin B resistance gene *HPH*, making it possible to follow the growth inside the plant using hyphal fluorescence. Doing so, we identified the rachis node as the flower tissue where growth of the Δ*fgp1* mutant was halted. No fluorescence from the Δ*fgp1* mutant was found in the rachis node (Figure 3C, red circle) nor was fluorescence detected beyond the rachis over the complete time period of two weeks. Growth of Δ*fgp1* was observed in other spikelet parts after three days (Figure 3C, white arrow). In contrast, fluorescence of the Δ*fgp2* mutant was found in the flower (arrow) as well as in the rachis node and beyond after three days (Figure 3D, red circle). The inability of the Δ*fgp1* mutant to penetrate the rachis node and the consequential pathogenicity loss might be caused solely by the lack of trichothecene production by the *fgp1* mutant. This inference is made because Δ*tri5* mutants of *F. graminearum*, lacking the gene for the first enzymatic step in toxin synthesis, are unable to produce trichothecene toxins *in planta* and also are stalled during infection at the rachis node [21].

### The Δ*fgp1* strains exhibit normal vegetative growth but show defects in asexual and sexual spore development

To determine whether the inability to cause disease could be correlated to a major growth or developmental deficiency, the growth characteristics of Δ*fgp1* strains were assessed on different media. Germination rate was assessed by placing freshly produced spores on PDA agar. Spores from WT PH-1 show almost complete (≥95%) germination after 8 hours, while Δ*fgp1* spores show a slight delay with complete (≥95%) germination observed after 12 hours (data not shown). On PDA and minimal medium, the deletion of *FGP1* in wild type results in a slightly reduced radial growth phenotype, which can be attributed to the delayed germination. Complementation of the Δ*fgp1* strain with the wild type *FGP1* gene restored the wild type growth phenotype. No growth differences were observed on complex solid media including V8, carrot or mung bean agar plates (data not shown).

**Figure 4:**
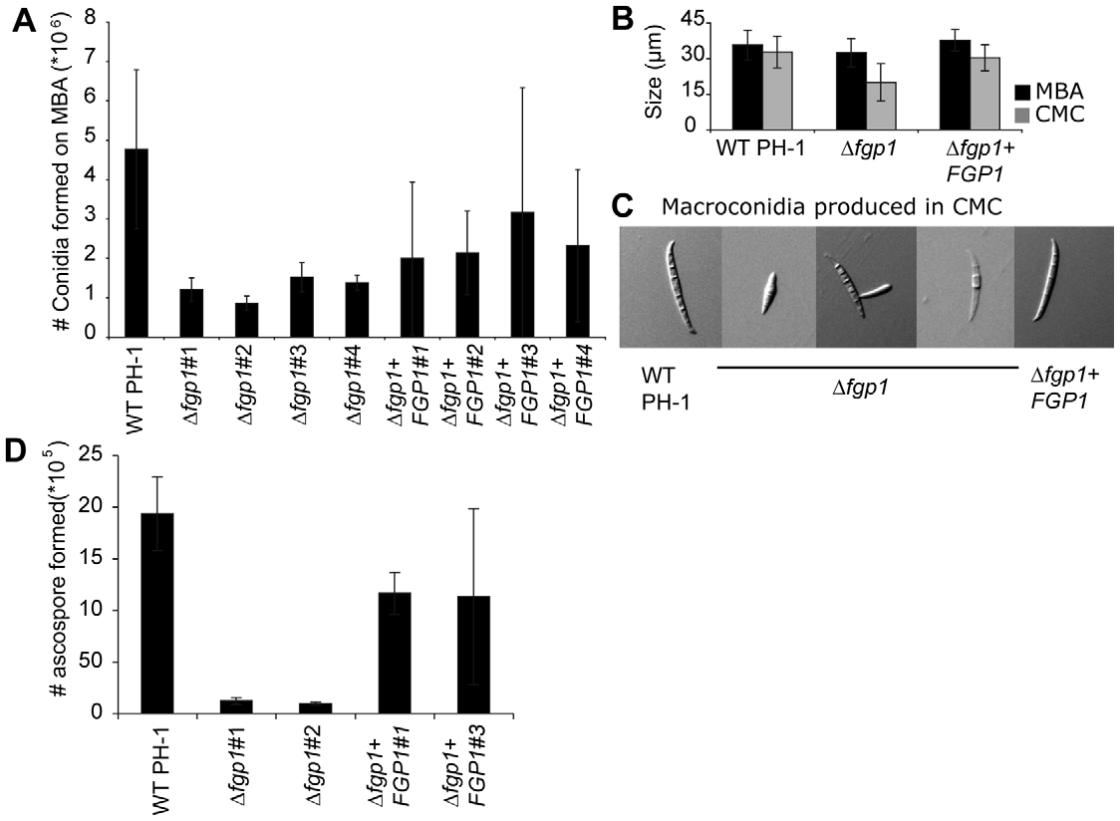
Fgp1 is involved in conidiogenesis as well as in ascospore formation. A) Macroconidia production was assessed in three experiments each in two replicas on mung bean agar (MBA) plates and counted in a haemocytometer. B) Average macroconidium length was determined by measuring 30 macroconidia of each strain. C) Photos of a representative wild type PH-1 macroconidium (left), three representative conidia formed by the *FGP1* deletion mutant (middle) and a representative *FGP1* complementation strain macroconidium (right). D) Ascospore production by wild type PH-1, two *FGP1* deletion mutants and two *FGP1* complementation strains was assessed in four replicas for each strain on carrot agar plates two weeks after the mycelium was knocked down using a 2.5% Tween-60 solution. Ascospores were counted with a haemocytometer.

**Figure 5:**
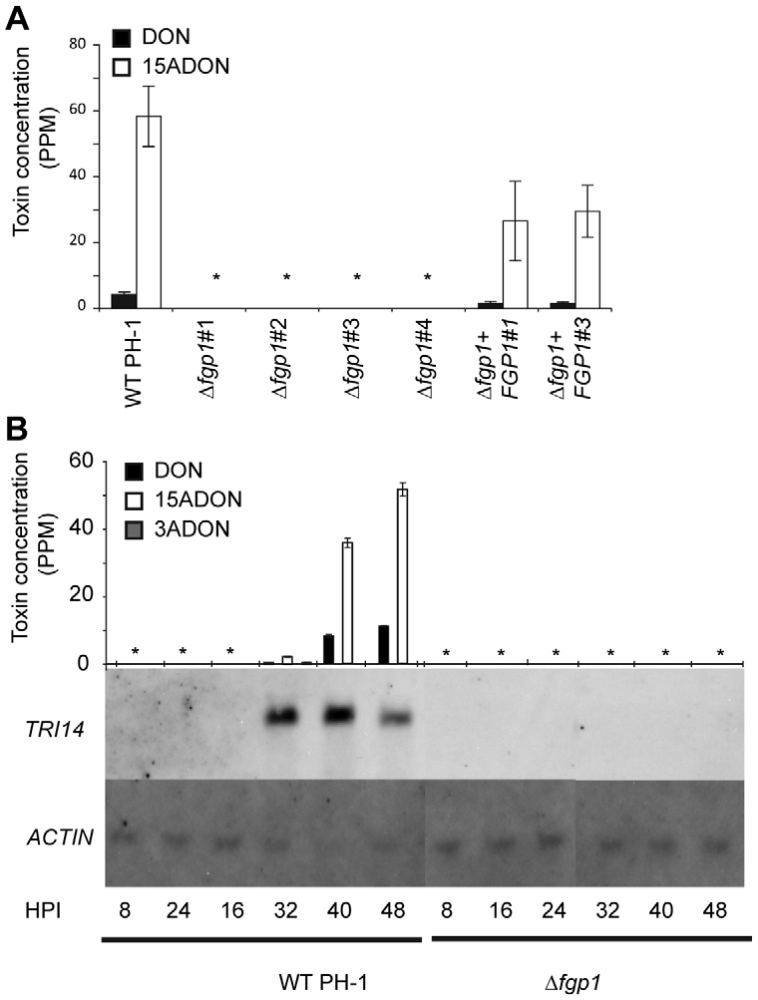
The *FGP1* deletion strain does not produce trichothecene toxins in putrescine medium. A) Histogram of toxin concentration (parts per million - ppm) of DON and 15-ADON measured in putrescine medium after one week of growth of wild type, four Δ*fgp1* strains and two complemented strains. Asterisks indicate no toxin was detected. B) Histogram of toxin concentration (parts per million - ppm) of DON, 15-ADON and 3-ADON measured in putrescine medium with samples taken at 8, 16, 24, 32, 40, and 48 hours post inoculation (HPI) with wild type PH-1 or a *FGP1* deletion strain. Detectable toxin concentrations are found at 32, 40 and 48 HPI in the wild type PH-1 but not in the *FGP1* deletion strain at any time point. Asterisks indicate no toxin was detected. A northern blot of RNA obtained from the same samples shows expression of the *TRI* gene, *TRI14*, after 32, 40 and 48 HPI in the wild type PH-1 but not in the *FGP1* deletion strain at any time point. The loading control gene *ACTIN* is expressed equally at all time points for both wild type and the *FGP1* deletion strain.

Fgp1 is required for full asexual spore formation as was observed for Sge1 in *F. oxysporum* [9]. During growth on mung bean agar (MBA), fewer (p≤0.05, Student's t-Test) macroconidia are formed in four independent Δ*fgp1* deletion strains compared to wild type (Figure 4A). Additionally, spores of the Δ*fgp1* strain are smaller during growth on mung bean agar (MBA) (p≤0.05, Student's t-Test) or in carboxymethylcellulose (CMC) (p≤0.01, Student's t-Test) (Figure 4B) or may exhibit precocious germination or appear not fully developed (three middle pictures, Figure 5C representative spores are shown). In the complementation strain, the quantity of spores is restored, albeit incompletely, still, the spores produced resemble wild type (Figure 4A, B and C, representative spores of wild type and complemented strain are shown). Using Calcofluor white and Hoechst staining, no defects in cell wall composition or nucleus quality were observed in the Δ*fgp1* strain (data not shown).

When strains were grown on carrot agar to induce perithecium and ascospore formation, it was observed that perithecia formed by the Δ*fgp1* strain are comparable to wild type (data not shown). Ascospore formation on the other hand was delayed by one week in the Δ*fgp1* strain and altogether only a few ascospores emerged from the perithecia. Cirrhi of wild type PH-1 and a *FGP1* complementation strain appear one week after the formation of the perithecia and consist of strings of ascospores emerging from perithecia (data not shown). Cirrhi of the *FGP1* deletion strains appear two weeks after the formation of the perithecia and contain only a few ascospores at the perithecial apex (data not shown). When the ascospores were harvested and counted, fewer ascospores (p≤0.01, Student's t-Test) were produced by the Δ*fgp1* strains compared to wild type (Figure 4D). This defect was restored in the complementation strains (Figure 4D). These observations indicate that *FGP1* is involved the developmental processes of conidiogenesis and ascospore formation although these processes are not fully abolished in the deletion mutant. As a consequence of this impaired conidiogenesis, the Δ*fgp1* strain probably displayed delayed germination on certain media. On the other hand, Fgp1 has no impact on normal vegetative growth.

Since the Fgp2 homolog Pac2 is involved in the sexual cycle of *Schizosaccharomyces pombe*, we also tested whether perithecium and ascospore formation was altered in the Δ*fgp2* strain. No major differences with respect to perithecium and cirrhi formation were noted when compared to wild type (data not shown).

### Fgp1 in *F. graminearum* is required for *TRI* gene expression and toxin production *in vitro*

Previous studies have shown that *F. graminearum* produces trichothecene toxins when grown on medium containing polyamine compounds [22] and that this medium induces the expression of genes involved in trichothecene production [5]. Since trichothecene toxins did not accumulate in wheat spikelets inoculated with Δ*fgp1*, we investigated whether deletion strains also are impaired in production of toxin *in vitro*. In order to do this, the wild type, four independent Δ*fgp1* strains and two independent complemented strains were inoculated into putrescine containing medium and after one week assessed for the presence of trichothecene toxins. Toxin was found in wild type cultures but no toxin was detected in the cultures of the Δ*fgp1* strains (Figure 5A). Toxin was also found in the cultures of the two complemented strains albeit to slightly lower levels than wild type (Figure 5A).

The lack of toxin production in the Δ*fgp1* strains could be due to the inability to express the genes from the *TRI* cluster. In order to test for this, a northern blot experiment was performed. Both wild type PH-1 and a Δ*fgp1* strain were grown in minimal medium containing the polyamine putrescine or in control minimal medium with NaNO_3_ as the sole nitrogen source instead of putrescine. Samples for RNA isolation were taken 8, 16, 24, 32, 40 or 48 hrs after inoculation and the RNA was used for gel electrophoresis and capillary blotting. Northern blots were hybridized with *TRI14*, a gene from the trichothecene biosynthetic cluster [23] or with a constitutively expressed actin gene used as the loading control. *TRI14* is expressed to high levels during growth in polyamine medium [24] and is therefore a good marker gene for the expression of the *TRI* cluster. No *TRI14* transcript was observed in the Δ*fgp1* mutant strain grown in putrescine medium at any time point in contrast to wild type PH-1 in which expression of *TRI14* was detected at 32, 40 and 48 h (Figure 5B). RNAs corresponding to *TRI14* were not detected in cultures grown in minimal medium for either wild type or Δ*fgp1* (data not shown). *Actin* transcripts were detected in all samples of wild type and Δ*fgp1*, indicating equal loading.

Analysis of the cultures filtrates of the different samples revealed that trichothecene toxins were present in the putrescine medium containing wild type PH-1 at 32 h and accumulated to higher levels at 40 and 48 h (Figure 5B). No toxin was detected for the Δ*fgp1* strain grown in putrescine medium at any time point nor in control medium, which is consistent with the previous toxin analysis experiment and the lack of *TRI14* gene expression. The Δ*fgp1* strain exhibits growth equal to wild type in putrescine medium (data not shown), suggesting that it likely can take up putrescine and use it as a nitrogen source but this does not lead to *TRI* gene expression. In minimal medium lacking any nitrogen source, growth of both wild type and the Δ*fgp1* strain was noticeably reduced and no toxin was detected (data not shown). This suggests that *TRI* gene expression is not triggered by the absence of a nitrogen source but rather that putrescine is a specific inducer. Clearly, Fgp1 is required for putrescine induced trichothecene production.

The full HPLC spectra of the wild type and Δ*fgp1* samples grown for 40 hours in putrescine medium were, besides used for trichothecene toxin identification, also inspected for other differences in metabolite profiles. Doing so, no significant peaks other than the peaks corresponding to DON and 15-ADON are missing in the spectrum from the Δ*fgp1* strain compared to the wild type (Figure S3). This suggests that Δ*fgp1* can still produce a majority of the other metabolites present in the spectrum and that it does not appear to be impaired in production of metabolites in general.

### A hyphal morphological change is observed in putrescine medium for both the wild type and the Δ*fgp1* strain

**Figure 6:**
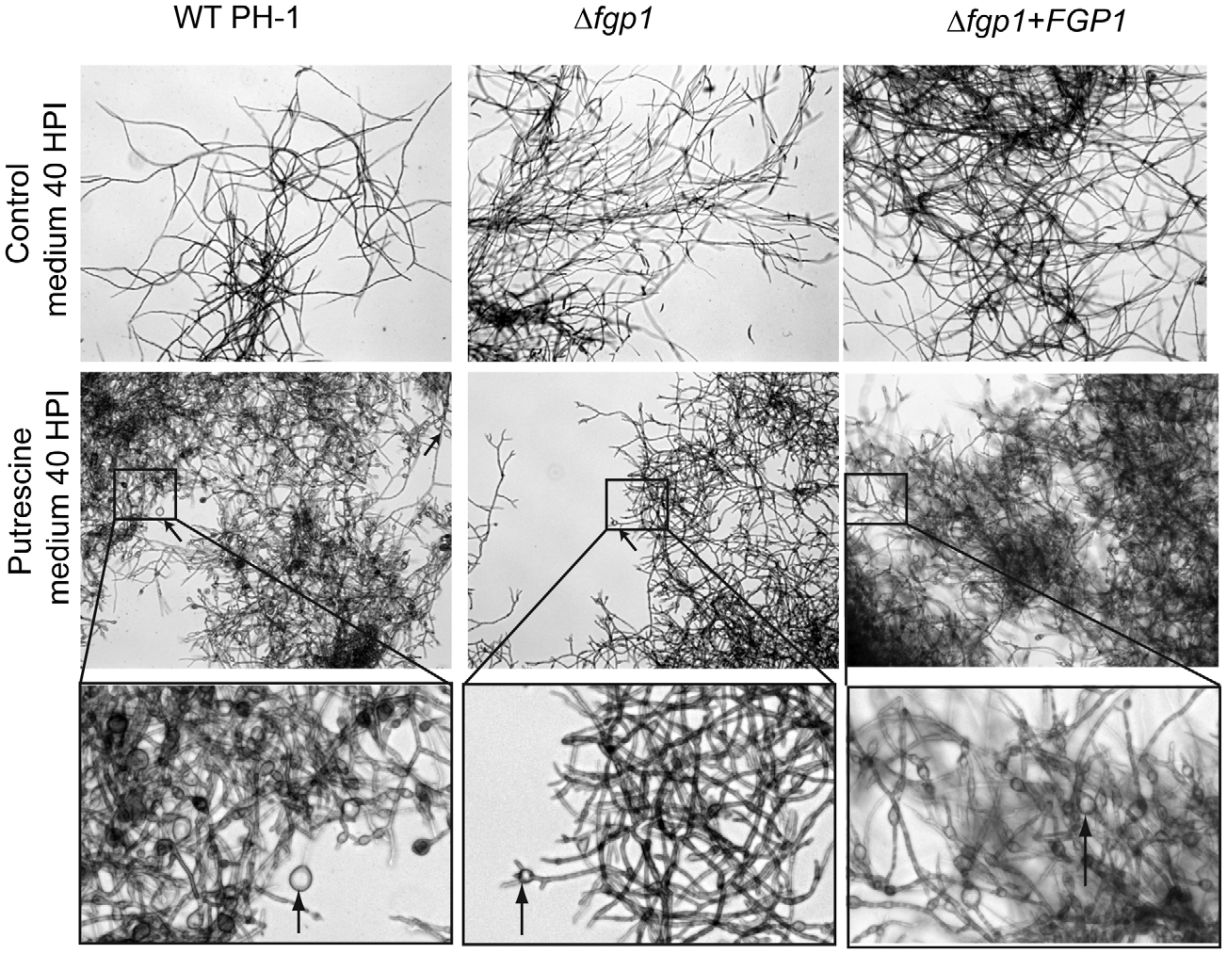
Vigorous hyphal swelling during growth in putrescine medium is seen to a lesser extent in Δ*fgp1*. Mycelium of wild type, a *FGP1* deletion strain and a complemented strain growing in control minimal medium (upper panel) or putrescine (middle panel) at 40 HPI. A representative area of the mycelium observed under the microscope is given for each strain. Mycelium of wild type PH-1 and the complemented strain show branching and numerous hyphae with bulbous structures when compared to mycelium grown in control minimal medium. Mycelium of the *FGP1* deletion strain also shows branching but fewer hyphae with bulbous structures. Greater magnifications of the boxed frames from the middle pictures (lower panel) show that in the mycelium of the wild type and complemented strains many bulbous structures are formed (left and right pictures in the lower panel). The mycelium of the Δ*fgp1* strain shows some bulbous structures but less when compared to with wild type and complemented strain (middle picture in the lower panel). Bulbous structures are indicated by arrows in the middle and lower panels.

To examine in more detail why *TRI* gene expression was not observed in the Δ*fgp1* strain, wild type PH-1, Δ*fgp1* and complemented strain were grown in putrescine medium and examined microscopically. In all strains, a distinct morphological change in hyphae was observed when cultures were grown in putrescine medium for 40 h compared to control medium. In control medium, hyphae display a uniform thickness over their entire length and grow in long branches in wild type, Δ*fgp1*, and complemented strain (Figure 6, upper panel). In putrescine medium, the wild type and complemented strains display hyphae that produce bulbous sub-apical structures, which are to some extent also observed in the Δ*fgp1* strain (Figure 6, center panel). In wild type and complemented strain, bulbous structures up to 21 µm in diameter are observed among numerous other, but fewer and smaller bulbous structures are observed in the Δ*fgp1* strain (Figure 6, lower panel). Another independent Δ*fgp1* and complemented strain displayed similar growth morphology as the Δ*fgp1* and complemented strain given in Figure 6.

To investigate whether this morphological change in the putrescine medium parallels the expression of *TRI* genes and trichothecene accumulation we also examined the cultures at the different time points. After 16 and 24 h, some hyphae begin to swell and form bulbous structures in both wild type and Δ*fgp1* strains (see arrows, Figure S4). These structures become more abundant at 32 h in the wild type but not in the Δ*fgp1* strain (see arrows, Figure S4). At 40 and 48 h, large bulbous structures appear in the wild type which are less apparent in the Δ*fgp1* strain (see arrows, Figure S4). This suggests that the formation of these bulbous structures might be involved in the production of trichothecene toxins and that somehow the Δ*fgp1* strain is less capable to form the same structures as in wild type.

To examine whether the bulbous structures observed in wild type are formed as a consequence of toxin production or whether the structures may accommodate toxin production, a Δ*tri6* mutant was grown in putrescine and control minimal medium. *TRI6* encodes a transcription factor that is absolutely required for the expression of the genes in the *TRI* cluster and for trichothecene synthesis [25]. The Δ*tri6* strain lacks detectable trichothecene accumulation in the putrescine medium (data not shown), but the hyphae show the same degree of hyphal swelling and bulbous structure formation as the wild type (data not shown). We conclude from this that the bulbous cells do not form in response to trichothecene synthesis because they appear to develop before transcription of the *TRI* genes, and the toxins themselves, can be detected. It remains unclear what the exact role this hyphal swelling plays in toxin synthesis and how Fgp1 might be involved in the facilitation of this process.

### Fgp1 regulates many genes during growth in putrescine medium and during infection

To investigate the underlying genetic basis for the differences in toxin accumulation and hyphal morphology seen in putrescine medium, RNA was extracted from wild type PH-1 (three independent samples) and Δ*fgp1* strains (three independent samples) after growing 40 h in putrescine and labeled for microarray experiments. Additionally, in order to find genes regulated by Fgp1 during *in planta* growth, RNA was extracted from wheat heads inoculated for 72 hours with wild type (three independent samples, three heads per sample with ten inoculated spikelets per head) or Δ*fgp1* (three independent samples, three heads per sample with ten inoculated spikelets per head) and labeled for microarray experiments. The number of genes showing a >2-fold difference in average expression between wild type PH-1 and Δ*fgp1* strains during growth on putrescine medium and during wheat infection were determined (Figure S5). Fgp1 appears to regulate gene expression positively as well as negatively during growth in putrescine medium and infection of wheat with hundreds of genes differentially expressed. In total, 654 genes show a >2.0-fold (P<0.05) lower expression in Δ*fgp*1 compared to wild type when grown in putrescine and 536 genes show higher expression in Δ*fgp*1 (Table S2). Additionally, 634 genes were ≥2-fold lower expressed (P<0.05) in the Δ*fgp1* strain compared to wild type during wheat infection and 39 genes were found >2 fold higher expressed (Table S3).

Some overlap is found between genes positively or negatively regulated by Fgp1 in putrescine medium and those regulated during wheat infection (91 and 3 genes, respectively (Figure S5)). Recently, a gene set considered to be expressed “*in planta* only” was described containing genes from *F. graminearum* exclusively expressed during plant infection but not in axenic cultures [26]. Out of the 369 genes from this gene set, 243 were found expressed in the experiments described here, either in wild type or the Δ*fgp1* strain grown in putrescine medium (45 genes) or in infected wheat (199 genes) (Table S4). Overlap was found between these 243 “*in planta* only” genes and genes lower or higher expressed in the Δ*fgp1* strain in putrescine medium or during wheat head infection (Figure S5). Altogether, Fgp1 regulates a remarkably large proportion of genes considered “*in planta* only”; 99 out of 244 or 41% were positively regulated and another nine (3.7%) were negatively regulated.

Fgp1 positively regulates thirteen “*in planta* only” genes in putrescine as well as in wheat heads. Among these thirteen are six genes from the trichothecene biosynthetic cluster (FGSG_03534, FGSG_03536, FGSG_03537, FGSG_03538, FGSG_03540 and FGSG_03543) as well as four other genes involved in trichothecene toxin synthesis or co-expressed with *TRI5* [24] (FGSG_00007, FGSG_01819, FGSG_07562 and FGSG_10397). Among the remaining three are FGSG_08079 a gene involved in butenolide synthesis, FGSG_08309 encoding an ABC transporter with orthologs in other fungi and FGSG_02120 for which there is no annotated function or ortholog found in any other fungi with a sequenced genome. Fgp1 negatively regulates one “*in planta* only” gene expressed in putrescine medium and during infection: FGSG_11033.

*TRI6* (FGSG_03536) encodes a transcription factor that regulates the expression of other *TRI* genes and genes involved in the isoprenoid biosynthesis pathway. Since *TRI6* and an ancillary transcription factor, *TRI10*, are both extremely down-regulated in the Δ*fgp1* strain, genes positively regulated by Tri6 and Tri10 might be expected to be expressed at lower levels in the Δ*fgp1* strain. Indeed, 56 and 62 genes expressed at lower levels in the Δ*fgp1* strain in putrescine and wheat head, respectively (Table S5), were also found to be expressed at lower levels in the Δ*tri6*/*tri10* strains 96 h after wheat inoculation. Among the common regulated genes are the ones involved in the isoprenoid biosynthesis pathway. This suggests that Fgp1 may assume a portion of its regulatory control by acting upstream of the Tri6 and Tri10 transcription factors.

### Fgp1 regulates expression of gene clusters and other genes encoding PKS or NPS proteins

**Figure 7:**
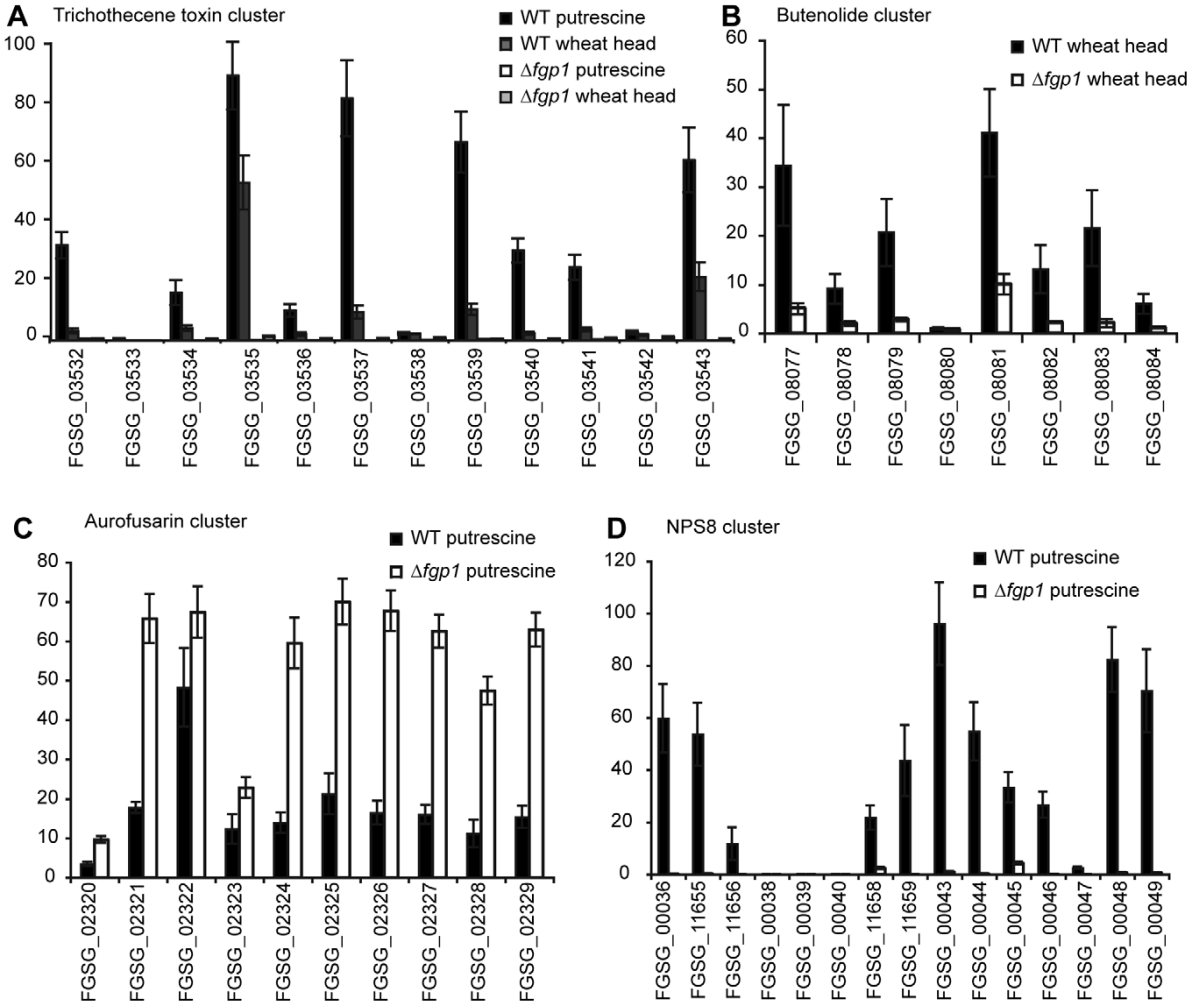
Fgp1 mediates expression of genes in several gene clusters. Histograms represent mean relative expression levels from three replicate experiments. Error bars represent standard deviation. A) Expression means for genes from the *TRI* cluster in wild type PH-1 grown in putrescine (black bars) or during wheat head infection (dark gray bars) and in a *FGP1* deletion strain grown in putrescine (white bars) or during wheat head infection (light gray bars). B) Expression means for genes from the butenolide biosynthetic cluster in wild type PH-1 during wheat head infection (black bars) and in a *FGP1* deletion strain during wheat head infection (white bars). C) Expression means for genes from the aurofusarin biosynthetic cluster in wild type PH-1 grown in putrescine medium (black bars) or for a *FGP1* deletion strain grown in putrescine medium (white bars). D) Expression means for genes from the NPS8 cluster in wild type PH-1 grown in putrescine medium (black bars) or a *FGP1* deletion strain grown in putrescine medium (white bars).

As co-regulated clusters of genes often are associated with secondary metabolite biosynthesis, expression of putative gene clusters from *F. graminearum* identified previously [27] was examined using the expression values obtained from the microarray experiment. Unlike in wild type, expression of all genes involved in trichothecene biosynthesis was absent or was greatly reduced in the Δ*fgp1* strain during growth in putrescine medium as well as during wheat infection (Figure 7A). This loss of expression was confirmed by the absence of *TRI14* transcript in the northern blot experiment (Figure 5) and lack of the trichothecene toxins produced by the Δ*fgp1* strain. In addition to the twelve genes from the *TRI* cluster, 19 of the 38 genes that showed co-expression with *TRI5* in agmatine medium [24] are also regulated by Fgp1, either during growth in putrescine medium or during wheat infection (Table S6). Fgp1 negatively regulates two of the 19 genes: FGSG_01832 and FGSG_03132. Five of the 17 genes that are positively regulated by Fgp1 are also regulated by Tri6 (Table S6).

Genes involved in butenolide synthesis, also reside in a cluster [28]. Other than FGSG_08077, FGSG_08078 and FGSG_08079, expression of genes from the butenolide cluster was not detected in putrescine medium for both wild type and Δ*fgp1*. However, during wheat infection expression of all genes from the cluster were detected on the microarray chip in wild type and in the Δ*fgp1* strain, albeit to a significantly lesser extent for the mutant (Figure 7B).

A cluster of genes involved in the production of the yellow or purple pigment aurofusarin [29] as well as genes comprising the non-ribosomal protein synthase 8 (NPS8) [24] gene cluster also appear to be regulated by Fgp1. In the Δ*fgp1* strain, the aurofusarin cluster genes are up-regulated when compared to wild type suggesting a role for Fgp1 in the negative regulatory control of this cluster (Figure 7C). The genes of the NPS8 cluster are highly down-regulated or not detected in putrescine medium in the Δ*fgp1* strain when compared to wild type (Figure 7D). For both gene clusters no gene expression was detected during wheat infection.

Fgp1 also regulates other genes that encode polyketide synthases (PKS) [30] and non-ribosomal protein synthases (NPS) [31] that may be involved in secondary metabolite synthesis. During wheat infection *NPS9* (FGSG_10990), *NPS12* (FGSG_11294), *NPS14* (FGSG_11395), *PKS7* (FGSG_13295) and *PKS15* (FGSG_04590) are expressed at lower levels in the Δ*fgp1* mutant compared to wild type. During growth in putrescine medium *NPS11* (FGSG_03245) is expressed at lower levels in the Δ*fgp1* mutant. Higher expression was observed for *NPS18* (FGSG_13783) in the Δ*fgp1* mutant during growth in putrescine medium. Transcriptome experiments therefore show that Fgp1 regulates gene clusters and genes predicted to encode secondary metabolite synthesis proteins and genes previously found solely expressed during plant infection.

### Comparative transcriptomics of Δ*sge1* from *F. oxysporum* and Δ*fgp1* from *F. graminearum* during vegetative growth and sporulation

To determine whether Fgp1 and Sge1 share common regulatory targets, a comparative transcriptional study was conducted during growth in culture. Both wild type strains *Fol*4287 and PH-1 as well as deletion strains Δ*sge1* and Δ*fgp1* from *F. oxysporum* and *F. graminearum*, respectively, were grown in complete medium (CM) for 48 h. RNA was extracted, reverse-transcribed into cDNA, labeled and hybridized to microarray chips. In *F. graminearum*, 119 genes show a ≥2 fold lower expression in the Δ*fgp1* strain on CM and 83 genes a higher expression compared to WT (Table S7). For *F. oxysporum*, 394 genes show a ≥2 fold lower expression in the Δ*sge1* strain on CM and 819 a higher expression compared to WT (Table S7). When the gene sets regulated by either Fgp1 or Sge1 were compared, only a few orthologous genes were found to be regulated by both Sge1 and Fgp1 in the two *Fusarium* species (Figure S6). A set of 16 down-regulated and eight up-regulated orthologous genes were found in both deletion strains (Table S8). Remarkably, eleven genes up-regulated in Δ*sge1* were found down-regulated in Δ*fgp1* and one gene up-regulated in Δ*fgp1* was found down-regulated in Δ*sge1* (data not shown). Altogether, the results from this comparative transcriptomic experiment suggest that Fgp1 and Sge1 regulate largely non-overlapping sets of genes.

Because of the effect of the Δ*sge1* mutation on sporulation in *F. oxysporum* it was noteworthy that there was differential regulation between wild type and the Δ*sge1* mutant for several genes known to effect sporulation in *Fusarium* and other fungi. *REN1* [32], an ortholog of *MEDUSA* [33] from *Aspergillus nidulans*, *ABA1*, an ortholog of *ABACUS* [34] from *A. nidulans* and FOXG_01756, an orthologs of FlbC [35] from *A. nidulans*, were among the genes expressed at lower levels in the Δ*sge1* mutant. The lower expression of these genes could account for the fewer number of spores produced by the Δ*sge1* mutant [9]. *F. graminearum* does not produce conidia in CM, so, in order to study these genes, we grew wild type and Δ*fgp1* in liquid carboxymethylcellulose (CMC) medium or mung bean agar (MBA), two media that induce sporulation. Quantitative PCR experiments were conducted using cDNA obtained from *F. graminearum* wild type and Δ*fgp1* and from *F. oxysporum* wild type and Δ*sge1* strains during growth in conidia inducing medium. We used the constitutive expressed gene *FRP1* [36] as a reference in both *F. oxysporum* and *F. graminearum*. The expression levels of *FRP1* proved not to be influenced by deletion of *SGE1* or *FGP1* (results not shown). The results showed that in *F. oxysporum* all three genes (*REN1*, *ABA1* and FOXG_01756) were expressed at lower levels in the Δ*sge1* strain, confirming the microarray results, but in the Δ*fgp1* strain of *F. graminearum*, only the expression of *ABA1* was significantly reduced during growth on MBA and CMC compared to wild type (Figure S7); the expression of *REN1* and FGSG_ 07052 (*FLBC* ortholog) were not significantly lower; both genes even show a slightly higher expression in the Δ*fgp1* strain compared to wild type when grown on CMC. However, the expression of FGSG_ 07052 was lower in the Δ*fgp1* strain compared to wild type when grown on CM (Figure S7). These results suggest that Sge1 regulates a number of sporulation genes and that Fgp1 only regulates Aba1 during conidia formation. This expression difference may be attributable to the difference in spores that are formed under these conditions: microconidia in *F. oxysporum* versus macroconidia in *F. graminearum*.

### Interchanging FGP1 and SGE1 between *F. graminearum* and *F. oxysporum* does not complement function

**Figure 8:**
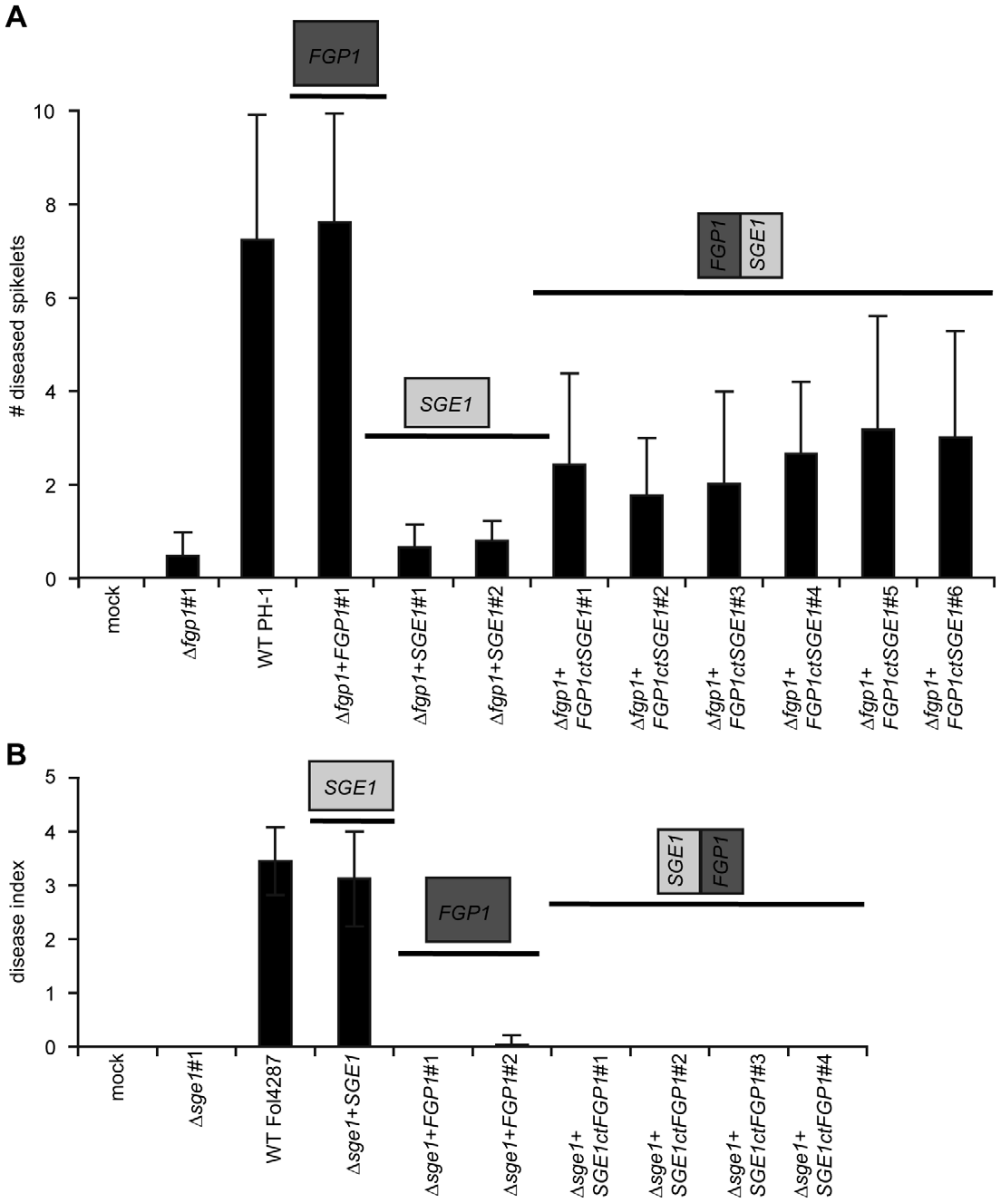
*FGP1* and *SGE1* are not functionally interchangeable between *F. graminearum* and *F. oxysporum*. *FGP1* can complement a *FGP1* deletion mutant but *SGE1* cannot. A chimeric *FGP1*/*SGE1* gene expressed from the *FGP1* promoter partially complements a *FGP1* deletion mutant. *FGP1* cannot complement a *SGE1* deletion mutation nor can a chimeric *SGE1*/*FGP1* gene expressed from the *SGE1* promoter A) Average number of diseased spikelets of 20–30 wheat plants, two weeks after point inoculation with H_2_O, a *FGP1* deletion mutant, wild type PH-1, one independent *FGP1* complementation strain, two independent *SGE1* complementation strains and six independent chimeric *FGP1*/*SGE1* strains. B) Average disease index of 20 tomato plants, three weeks after inoculation with H_2_O, a *SGE1* deletion mutant [9], wild type *Fol* strain 4287, the original *SGE1* complementation strain [9], two independent *FGP1* complementation strains and four independent chimeric *SGE1*/*FGP1* strains.

To study the functional conservation of *FGP1* and *SGE1*, the two genes were introduced in the opposite species in order to test whether they can take over each other's function. Doing so, *SGE1* was introduced into the Δ*fgp1 F. graminearum* strain and *FGP1* into the Δ*sge1 F. oxysporum* strain. Two independent transformants that carry the *SGE1* complement in the Δ*fgp1* strain in *F. graminearum* were obtained and were used to inoculate wheat spikelets. In contrast to the transformants harboring the *FGP1* complement, both *SGE1* complements were unable to restore disease causing ability on wheat heads (Figure 8A, bars below the *FGP1* box and *SGE1* box, respectively). This suggests that *SGE1* has diverged from *FGP1* in such way that it is unable to restore its function.

This observation led to the hypothesis that the functional specificity of the protein may be present in the highly diverged C-terminal portion of the protein. To test this, combinations of the two genes from *F. oxysporum* and *F. graminearum* were made and transformed into a Δ*fgp1* strain of *F. graminearum* and into a Δ*sge1* strain of *F. oxysporum*. A combination consisting of the N-terminal portion of Fgp1 (amino acids (aa) 1–219) and the C-terminal of Sge1 (aa 220–330), expressed from the native *FGP1* promoter was used to complement the Δ*fgp1* strain. The strains obtained showed less spread (p≤0.01, Student's t-Test) and disease in inoculated wheat spikelets after two weeks compared to the full length *FGP1* complements but more disease than complements with full length *SGE1* (Figure 8A, bars below the *FGP1*/*SGE1* box). This suggests that the highly diverged C-terminus of Fgp1 is required for full function in *F. graminearum*. Reintroduction of the complete wild type gene *FGP1* of *F. graminearum* (Figure 8B, bars below the *FGP1* box) used to complement the Δ*fgp1* strain into a Δ*sge1* strain of *F. oxysporum* failed to restore pathogenicity towards tomato in contrast to complementation with full length *SGE1* (Figure 8B, bars below the *SGE1*/*FGP1* box). The combination of the N-terminal domain of *SGE1* (encoding aa 1–218) and the C-terminal domain of *FGP1* (encoding aa 219–342) expressed from the native *SGE1* promoter also failed to complement the mutant phenotypes in the Δ*sge1* strain. This suggests that the diverged C-terminal portions of the genes are critical for their function in the species of origin.

## Discussion

In this study, the Wor1-like protein Fgp1 from *F. graminearum* was demonstrated to greatly influence plant infection and trichothecene mycotoxin production. The Fgp1 ortholog Wor1 in *C. albicans* also regulates pathogenicity by controlling dimorphic switching essential for mammalian infection. By analogy we hypothesize that Fgp1 and other orthologs like Sge1 (*F. oxysporum*) and Reg1 (*Botrytis cinerea*) in phytopathogenic fungi may also act as master regulators of plant infection. Deletion of the *WOR1* orthologs in these fungi does not cause obvious vegetative growth defects yet the deletions appear to lock the mutants in a nonpathogenic state. Since the diseases caused by these fungi are vastly different in their mechanisms of infection, pathogenesis and tissue- and host specificity, the orthologous Wor1 regulatory proteins must also have evolved to regulate these unique aspects of their disease causing ability.

### Fgp1 regulates the production of trichothecene toxins

This study is the first to demonstrate that a Wor-1 like protein has the ability to control the expression of genes for mycotoxin biosynthesis and well as other gene clusters for synthesis of fungal secondary metabolites such as aurofusarin and butenolide. Fgp1 positively regulates the *TRI* cluster but negatively the *AUR* cluster responsible for aurofusarin production. This phenotype is also seen in a heterochromatin protein encoding gene deletion mutant of *F. graminearum*, Δ*hep1* [37]. The similarity in phenotype between Δ*fgp1* and Δ*hep1* could suggest that Fgp1 regulates chromatin modification too. Trichothecene toxins produced by *F. graminearum* are central to its pathogenicity to wheat [21]. These toxins normally are synthesized during pathogenic growth and are induced within specific plant parts [3]. As trichothecene biosynthesis is strongly under the control of Fgp1, especially through the transcription factor genes *TRI6* and *TRI10*, the protein must have evolved to regulate the genes for mycotoxin synthesis that are peculiar to this disease. A morphological change that accompanies trichothecene accumulation and gene expression is noticeably altered in the Δ*fgp1* mutant, suggesting that Fgp1 may be involved in a morphological change required for pathogenicity. A similar morphological change occurs during infection of wheat; *in planta*, *F. graminearum* forms thickened hyphae and coralloid structures that resemble the bulbous hyphae that are observed in putrescine medium [38]. This morphological phenomenon has been associated with toxin biosynthesis gene expression *in planta* in various studies [38], [39], [40], however, the necessity of the morphological change for toxin accumulation remains to be determined.

### Fgp1 regulates many genes during different growth conditions

Using microarray analysis many putative downstream targets of Fgp1 were identified. Among these are several pathogenicity related genes [26], including ones previously described as infection specific, such as genes from the *TRI* cluster and others involved in toxin production [24]. We also found many putative downstream targets of Fgp1 and of its *F. oxysporum* counterpart Sge1 when grown on CM. Both proteins show a high degree of specificity towards their putative targets as very little overlap was found between orthologous genes in the two species regulated by either Fgp1 or Sge1. Additionally, of the hundreds of genes that are regulated by Δ*fgp1* in putrescine medium and during wheat infection, only eight are also regulated by Fgp1 during growth in CM (Table S9), suggesting that Fgp1 regulates specific sets of genes during toxin induction conditions (putrescine or wheat head) or during growth in rich, toxin non-inducing medium (CM).

A few target genes of Sge1 and Fgp1 identified in this study and confirmed by quantative PCR analysis, are involved in sporulation and some may also play a role during pathogenicity. For example, Sge1 regulates the expression level of *REN1*, a conserved gene required for adherence, biofilm formation and virulence in *A. fumigatus* [41] and absolutely required for microconidia formation but not for pathogenicity in *F. oxysporum* [32]. The lower expression level of *REN1* in Δ*sge1* might explain, at least partly, the lower number of microconidia produced in CM. *F. graminearum* does not produce microconidia under any condition tested and perhaps as a consequence, no difference in *REN1* expression is observed between wild type and Δ*fgp1* strains during macroconidia formation. Sge1 also regulates the expression level of *ABA1*, another gene involved in conidium formation. In *F. oxysporum* f. sp. *melonis*, a pathogen of melon, Aba1 regulates production of both micro- and macroconidia and is required for full virulence. The Δ*aba1* mutant of this strain shows delayed pathogenicity towards melon probably due to fewer spores produced inside the xylem vessels (personal communication Dr. Tsutomu Arie, Tokyo University of Agriculture and Technology, Japan). In addition to sporulation, the Aba1 ortholog in *Penicillium marneffei* is involved in the dimorphic switch [42] and in *A. fumigatus* in autolysis and cell death [43]. The expression levels of *ABA1* of *F. graminearum* are also regulated by Fgp1 during conidia formation but not during growth in putrescine medium or infection of wheat heads. The role of *ABA1* during infection is therefore still elusive. Another gene known to control conidia formation in fungi that is regulated by Sge1 is FlbC. In *A. nidulans*, FlbC regulates the developmental processes of conidia formation and sexual fruiting and is required for normal vegetative growth [35]. To the contrary, we found that Fgp1 does not regulate the expression of *FLBC* of *F. graminearum* during conidia formation. Instead we found that expression of *FLBC* is regulated by Fgp1 during growth on CM and putrescine medium. Interestingly, FlbC in *F. graminearum* is absolutely required for wheat infection but not for toxin production [44]. How FlbC regulates virulence independent of toxin production and whether and how *FLBC* is regulated by Fgp1 during wheat infection is not yet known. *FLBC* is an interesting candidate gene to investigate further regarding its role in pathogenicity and its putative function downstream of both Fgp1 and Sge1.

A transcription factor not involved in sporulation but nevertheless regulated both by Fgp1 and Sge1 during growth on CM is DAL81. Dal81 is a general activator of nitrogen metabolic genes in yeast, including those for γ-aminobutyrate (GABA) [45]. Its exact role in *Fusarium* species is not yet known but this transcription factor (FGSG_02068) is required for toxin production in *F. graminearum* but, paradoxically, apparently not for virulence [44].

### Possible functional DNA-binding properties of Sge1 and Fgp1

Whether Fgp1 and Sge1 are transcription factors and bind DNA directly is not known. Their conserved N-terminal regions contain putative DNA binding domains, previously called the GTI1/PAC2 domain [15], [16] but recently renamed the WOPR box (Wor1, Pac2 and Ryp1) [18]. This motif consists of two globular peptide domains: WOPRa and WOPRb. Via these two domains Wor1 is able to bind a 14-bp DNA sequence and thereby activates its target genes and itself via a positive feedback loop [18]. Of the fourteen base pairs, the ones located in positions 6 through 14 (TTAAAGTTT) are absolutely required for binding. By scanning the upstream regions of both *FGP1* and *SGE1*, variations to the *WOR1* motif were found 557 upstream of the ATG of *FGP1*; TTAAAGTTC and 644 bp upstream of the ATG of *SGE1*: TTAACGCTT. Whether these domains in the promoters of *FGP1* and *SGE1* are DNA binding sites required for *FGP1* and *SGE1* expression is unknown. However, a search conducted for these patterns in upstream regions in both the genomes did not unveil any enrichment for this pattern in the genes found up- or down-regulated by either Sge1 or Fgp1 (data not shown). Likewise, a specifically conserved DNA pattern was not found upstream of genes regulated by Fgp1 or Sge1 under different conditions using search engines like RSAT [46].

### Presence of WOR1-like genes in fungi

The evolution of the Wor1-like proteins involved a duplication of the ancestral *Wor1* like gene sequence prior to the divergence of the yeast -like and filamentous ascomycetous fungi. The duplication resulted in paralogous genes (*FGP1*/*WOR1* and *FGP2*/*PAC2*) that apparently have evolved quite different regulatory functions. While *FGP2*/*PAC2* orthologs have been shown to be dispensable for pathogenicity, several studies now have established a role for *FGP1*/*WOR1* in pathogenicity for a surprisingly diverse array of fungal pathogens of both plants and animals [9], [10], [11], [12]. The function of *FGP1*/*WOR1* however, clearly extends beyond its role in pathogenicity since transcriptome studies demonstrate its regulatory control over many other functions such as reproduction and secondary metabolism. *FGP1*/*WOR1* and *FGP2*/*PAC2* orthologs also are found in strictly non-pathogenic fungi like *Podospora anserina* (data not shown); however, interestingly, in the non-pathogenic fungus *Neurospora crassa* only the *FGP2*/*PAC2* ortholog is present.

Unlike the N-termini, the divergent glutamine-rich C-termini of *FGP1/WOR1* genes in *Fusarium* likely allow specificity of regulatory control that has evolved independently in each species. Indeed the sets of genes regulated by orthologs *FGP1* and *SGE1* in *F. graminearum* and *F. oxysporum*, respectively, show little overlap and swapping experiments indicate the function of the C-terminal domains is largely specific to the species of origin.

### Regulation of WOR1-like genes

How *WOR1/FGP1* genes are regulated themselves is still elusive although the mitogen activated protein kinase (MAPK), as well as the protein kinase A (PKA) pathway could be involved. In *B. cinerea*, *REG1* expression levels are regulated by two mitogen activated kinases: BcSAK1 and Bmp3 [10]. For *SGE1*, higher expression levels (±5-fold) are observed during *in planta* growth compared to growth in axenic culture [9], which might indicate that *SGE1* is regulated through expression levels too, but for *FGP1* no significant differences in expression levels were observed during the conditions tested (data not shown). In *F. oxysporum* and *F. graminearum*, both mitogen activated kinases are required for pathogenicity [47], [48] of which the Δ*gpmk1* mutant in *F. graminearum* lacks the ability to form bulbous infection hyphae *in planta* [38] which might indicate that this strain is also defective in production of the bulbous hyphea in putrescine medium. In *C. albicans*, Wor1 phosphorylation and subsequent activation is believed to be performed by Tpk2, a subunit of the PKA [19]. The conserved phosphorylation site of the Wor1-like proteins resembles a PKA site, making it likely to be phosphorylated by PKA. But whether Sge1 or Fgp1 are also phosphorylated and which kinase may be responsible for that is still unknown. However, a PKA mutant in *F. oxysporum* (Δ*focpkA*) is also impaired in root penetration and virulence [49].

In *C. albicans*, Wor1 negatively regulates Efg1 transcription levels in opaque cells directly and indirectly via Czf1 and is itself regulated by Wor2 [18], [50]. The conserved *EFG1* ortholog in *Fusarium* species is called *STUA* and has been studied in *F. graminearum* (*FgSTUA*) [51] and *F. oxysporum* (*FoSTUA*) [52]. Czf1 and Wor2 have no conserved ortholog in *Fusarium* species as sequences homologous to these genes cannot be located using low stringency BLAST searches of the *Fusarium* genomes. The expression level of *FGP1* seems to be negatively regulated in *F. graminearum* by StuA. During growth of the Δ*fgstuA* mutant in CMC and in a two-stage toxin induction medium levels of *FGP1* transcripts are ±10 and ±100-fold higher compared to wild type. During growth of the Δ*fgstuA* mutant on wheat head, on the other hand, no significant difference in *FGP1* levels were observed [51]. In contrast, *FgSTUA* or *FoSTUA* expression levels are not significantly different from wild type in Δ*fgp1* and Δ*sge1* mutants, respectively ([9] and data not shown). The Δ*fostuA* mutant is still able to infect its host but the Δ*fgstuA* mutant is impaired in pathogenicity and toxin production [51]. In both *F. graminearum* and *F. oxysporum*, StuA is involved in conidia formation [51], [52]. Overall, the Δ*fgstuA* demonstrates a more severe phenotype than the Δ*fgp1* mutant with greatly reduced vegetative growth and spores almost entirely absent [51]. These observations suggest that regulation of *STUA* and *FGP1* in *Fusarium* species occurs differently than their orthologs *WOR1* and *EFG1* in *C. albicans*.

In-depth phosphorylation experiments and expression studies of *FGP1* and *SGE1* with different mutant strains will be needed to identify other putative upstream activation factors. Additional work also will be required to fully understand the divergent roles of the N- and C- terminal domains on protein function and target specificity. Lastly, further investigations into the genome-wide impact of Fgp1 and Sge1 regulation on cell homeostasis, spore development and host infection will be needed to grasp a better understanding of this very interesting protein family.

## Material and Methods

### Strains, plants and culture and plant infection conditions

The fungal isolates used in this study are the sequenced strains of *F. graminearum* PH-1 and *F. oxysporum* f.sp. *lycopersici* (*Fol*) strain 4287. Also used were the *Fol* Δ*sge1* strain *SGE1*KO4 and complementation strain *SGE1*com79 reported earlier [9]. All fungal strains were kept at −80° and revitalized on potato dextrose broth plus agar (PDB and Bacto agar, Difco). *F. graminearum* strains were grown for five days in carboxymethylcellulose (CMC) medium and *F. oxysporum* in rich complete medium (CM) for macroconidia and microconidia production, respectively. *Agrobacterium tumefaciens* EHA105 [53] used for *Agrobacterium* mediated transformations was grown in LB containing 20 µg/ml rifampicin and at 28°C.

The wheat varieties “Norm” and “Bobwhite” and the wilt susceptible tomato variety “Bonny Best” (Reimer Seeds, North Carolina USA) were used for plant infection studies. Wheat pathogenicity assays were performed with cultivar “Norm” using point inoculation [54]. Pathogenicity was scored two weeks after inoculation by counting the number of infected spikelets. Wheat infection used for microarray studies were performed with cultivar “Bobwhite” using point inoculations of 10 spikelets in the head. 72 hour after inoculation, anthers were cut off and the infected spikelets were detached from rachis, collected and frozen until RNA processing [25]. Tomato infections were performed with two-week-old seedlings sown in vermiculite and given 20-20-20 NPK fertilizers after one week. Seedlings were inoculated using the root dip method and disease was scored as described previously [9].

### Construction of gene replacement and complementation constructs

In order to generate deletion constructs of *FGP1* and *FGP2*, PCR was used to amplify the up- and down- stream sequences of each gene using primers 2 & 3 and 6 & 7 (*FGP1*) and primers 9 & 10 and 12 & 13 (*FGP2*) (Table S10). PCR products were ligated into plasmid pPK2*hphgfp* [20] after both product and plasmid were digested using the appropriate restriction enzymes. The upstream flanks of *FGP1* and *FGP1* were cut with *Kpn*I and *Pac*I and the downstream flanks with *Hind*III and *Xba*I. For *FGP1*, first the upstream flank was ligated into the plasmid and then the downstream flank. For *FGP2*, first the downstream flank was ligated into the plasmid and then the upstream flank.

A *FGP1* complementation construct was generated using PCR and primers 14 & 15 (Table S10), which contain *Kpn*I and *Eco*RI restriction sites, and ligated in pGEMT-easy (Promega) and sequenced. A correct product was ligated into plasmid pRW1p [55], which was cut using the same enzymes: *Kpn*I and *Eco*RI. The complementation constructs for chimeric *FGP1* and *SGE1* genes were prepared using the complementation constructs for both *SGE1* and *FGP1* in plasmid pRW1p. The combination of the N-terminal *SGE1* with the C-terminal *FGP1* was amplified using primers 16 & 17 and 18 & 19 (Table S10). The combination of the N-terminal *FGP1* part and the C-terminal *SGE1* part was amplified using primers 20 & 21 and 22 & 23 (Table S10). PCR products were ligated in pGEMT-easy and sequenced. Correct N-terminal *SGE1* and C-terminal *FGP1* products were cut from pGEMT-easy using enzymes *Bgl*II and *Xba*I and *Xba*I and *Pvu*II, respectively. Correct N-terminal *FGP1* and C-terminal *SGE1* products were cut from pGEMT-easy using enzymes *Adh*I and *Xba*I and *Xba*I and *Bgl*II, respectively. Plasmid pRW1p*SGE1* [9] was cut using enzymes *Bgl*II and *Pme*I. Using a three-point ligation strategy, the N-terminal *SGE1* and C-terminal *FGP1* products were ligated into plasmid pRW1p*SGE1* cut using enzymes *Bgl*II and *Pme*I and the N-terminal *FGP1* and C-terminal *SGE1* products were ligated into plasmid pRW1p*FGP1* cut using enzymes *Bgl*II and *Adh*I.

For transformations of *F. graminearum*, a neomycin resistance cassette was ligated into the different pRW1p plasmids. This neomycin cassette was previously cut from pSM334 [56] using the flanking *Xba*I site and ligated into plasmid pAG1 [57] cut with *Xba*I. Subsequently, the neomycin cassette was cut from pAG1-Neo with *Bam*HI and ligated into pRW1p cut with the same enzyme.

### Fungal transformations

*Agrobacterium* mediated transformation used for *F. oxysporum* was performed as described previously [58]. *Agrobacterium* mediated transformation used for *F. graminearum* was performed as described previously [29] with the following alterations: A different *A. tumefaciens* strain was used (EHA105), filters containing resistant colonies were not transferred to fresh selection plates but instead an agar plug containing a drug resistant colony was placed in liquid CMC medium and after two days of growth, spores were filtered through one layer of sterile miracloth and plated onto PDA plates containing cefoxitin (300 µg/ml) and hygromycin or geneticin (150 µg/ml). Single spore colonies were subsequently transferred to a fresh PDA plate and mycelial plugs were stored at −80°C. Deletion mutants were tested by PCR and Southern analysis. Transformants made to complement the different deletion strains were checked by PCR for presence of the inserted construct (data not shown) and strains containing an insertion of the gene of interest were used.

### Southern blotting

DNA was extracted using the CTAB protocol [59] and 5–10 µg was used for restriction and loaded for gel electrophoresis. Transfer of DNA to HyBond N+ (GE Health Care) was performed using standard alkaline procedures according to the manufacturer's protocol. Probes for *FGP1* and *FGP2* were amplified by PCR using primers 24 & 25 and 26 & 27, respectively (Table S10). Probe hybridization and detection was performed using an AlkPhos kit and CDP-Star chemiluminescent solution (GE Health Care) according to the manufacturer's protocol.

### Analysis of conidiogenesis, conidia germination and ascospore formation

For quantification of microconidia, three independent experiments were performed, each with two replicates Microconidia were harvested after five days of growth in 100 ml CMC medium and 1 ml of 2*10^4^ spores/ml were inoculated into 25 ml of fresh CMC medium. Alternatively 50 µl of a 1*10^6^/ml spore suspension were inoculated into 5 ml of CMC in a 24-deep well plate (1*10^4^ spores per well) and incubated for 5 days. the third method used was to count spores produced on mung bean agar (MBA), using 2 µl of a 1*10^6^/ml spore suspension (2*10^3^ spores) to inoculate a mung bean agar plate. After one week of incubation, two ml of water was added to the plate and spread over the mycelium to collect spores. One ml of the spore suspension was pipetted into a tube and spores within a volume of 10 µl were counted using a haemocytometer.

To assess the spore length, 40–75 spores produced in either CMC or on MBA were placed under a Nikon Eclipse 90i microscope and their length was measured. Perithecium formation was analyzed using the modified carrot agar method as described previously [60] in three replicas. To count the amount of ascospores, two ml of water was spread over the perithecia to collect ascospores. One ml of the ascospore suspension was pipetted into a tube and spores within a volume of 10 µl were counted using a haemacytometer.

### Toxin analysis

For *in vitro* toxin analysis, conidia (1*10^4^ sp/ml) of each strain were inoculated in six wells containing 2 ml of putrescine medium (30 g/l sucrose, 1 g/l KH_2_PO_4_, 0.5 g/l MgSO_4_, 0.5 g/l KCl, 0.8 g/l putrescine, 2 ml/l FeSO_4_*H_2_O solution (5 mg/ml) and 200 µl trace elements (50 g/l citrate, 50 g/l ZnSO_4_*7H_2_O, 2.5 g/l CuSO_4_*5H_2_O, 0.5 g/l H_3_BO_3_, 0.5 g/l NaMoO_4_*2H_2_O, 0.5 g/l MnSO_4_*H_2_O) and grown for 1 week in the dark at 25°C. For microscopy and the time series experiment, flasks containing 25 ml of putrescine medium or minimal medium (30 g/l sucrose, 1 g/l KH_2_PO_4_, 0.5 g/l MgSO_4_, 0.5 g/l KCl, 2 g/l NaNO_3_, 2 ml/l FeSO_4_*H_2_O solution (5 mg/ml) and 200 µl trace elements (50 g/l citrate, 50 g/l ZnSO_4_*7H_2_O, 2.5 g/l CuSO_4_*5H_2_O, 0.5 g/l H_3_BO_3_, 0.5 g/l NaMoO_4_*2H_2_O, 0.5 g/l MnSO_4_*H_2_O) were inoculated with 2000 spores/ml and grown in the dark at 25°C with shaking 150 rpm. For each time point, the filtrate was collected by passing it through one layer of miracloth and 250 µl of culture filtrate was placed in a glass vial and lyophilized. *In planta* trichothecene analysis was performed by placing the inoculated spikelet in a glass vial and measuring its weight. Determination of DON, 3ADON and 15ADON concentration per unit mass in the vials was performed as described earlier [54].

### RNA extraction, northern blot and reverse transcriptase-QPCR

RNA was extracted using Trizol (Invitrogen) according to manufacturer's protocol with an alternative precipitation step using ½ volume of isopropanol and a ½ volume of salt solution (0.8 M Sodium Citrate, 1.2 M NaCl). RNA was extracted from mycelium growing in complete medium for 48 hours in the dark at 25°C with shaking 150 rpm, from CMC grown cultures in a 24 well plate in 12 h light cycle at 25°C with shaking 150 rpm, from MBA grown cultures in 12 h light cycle at 25°C and from putrescine and minimal medium grown cultures for each time point as described above. Mycelium from cultures was harvested by filtration over one or two layers of miracloth, washed with water and frozen in liquid nitrogen. Mycelium was then lyophilized and ground in a mortar and pestle prior to Trizol extraction. RNA was also isolated from inoculated wheat spikelets, which were harvested, frozen in liquid nitrogen and ground in a mortar and pestle prior to Trizol extraction.

For northern blotting 15 µg RNA in loading buffer (0.5× 3-(N-morpholino) propanesulfonic acid buffer (MOPS), 1 M deionized glyoxal, 50% DMSO) was loaded onto a 1× MOPS 1% agarose gel. The RNA was subsequently blotted onto Hybond-N+ (GE Health Care) using a capillary blotting protocol provided by the manufacturer with 20× SSC as transfer buffer. The *TRI14* probe was amplified using primers 28 & 29 (Table S10) followed by *Bam*HI digestion and gel purification of the 897 bp fragment containing the portion of *TRI14* downstream of the preditcted intron. The *ACTIN* probe was amplified using primers 30 & 31 (Table S10). Probe hybridization and detection was performed using the AlkPhos kit and CDP-star chemiluminescent solution (GE Health Care) according to the manufacturer's protocol.

RNA cleanup was done using the RNeasy Mini Kit (Qiagen) prior to reverse transcriptase or microarray labeling. RNA labeling reactions were performed according to the standard Affymetrix protocols. The putrescine and minimal medium samples were hybridized to the Affymetrix *F.* graminearum GeneChips [61] and the complete medium and wheat infection samples were hybridized to an updated Affymetrix nine fungal plant pathogen GeneChip (www.plexdb.org). Hybridizations were performed at the BioMedical Genomics Center of the University of Minnesota.

RNA (2 µg) was treated with DNase (Invitrogen) and used for RT-PCR with SuperScript III Reverse Transcriptase (Invitrogen) according to manufacturer's protocol. The cDNA obtained by different methods was used as template for Quantitative PCR (qPCR), which was performed in two replicates with DyNamo™ SYBR® Green qPCR (Finnzymes) using a DNA-Engine Peltier thermal cycler (BioRad) equipped with a Chromo4™ real-time PCR detector and MJ Opticon Monitor™ analysis software. To quantify mRNA levels of genes of interest the ΔCt method was used. Primers used for constitutively expressed *FRP1* genes (primers 32–35) and the respective sporulation genes (primers 36–47) are listed in Table S10.

### Microscopy

Fungal infection in spikelets was monitored using an Olympus SZX16 Research Stereo Microscope and perithecia and cirrhi formation was observed using an Olympus SZX12 Research Stereo Microscope. Hyphal morphology of fungal strains growing in putrescine and control minimal medium was observed using a Nikon Eclipse 90i microscope.

### Microarray data analysis

CEL files were imported in Refiner 5.3 software (Expressionist) and RMA preprocessing was applied. Signal values (p-value 0.04) obtained in the Analyst software (Expressionist) were normalized to the median. Fold-expression filters were applied as described in the results. The probe sets and the corresponding probe descriptions from the *F. graminearum* GeneChips were converted from the annotation of the FG1 assembly to the FG3 assembly using the *Fusarium graminearum* database of MIPS (http://mips.helmholtz-muenchen.de/genre/proj/FGDB/) in order to compare the experiments [62]. Probe sets on the nine fungal plant pathogen genome array GeneChips was designed based on the FG3 assembly for *F. graminearum* and the FO2 assembly for *F. oxysporum* (Fusarium Comparative Sequencing Project, Broad Institute of Harvard and MIT (http://www.broadinstitute.org/)). For the experiments with both *F. graminearum* and *F. oxysporum* grown in CM, a *F. oxysporum* ortholog was queried by BLAST [17] for every *F. graminearum* gene showing altered expression. In order to establish whether a gene was conserved in both *F. oxysporum* and *F. graminearum*, we searched for the respective orthologs using BLAST and a hit was considered a full ortholog at a bit score of >200. Data and CEL files for microarray experiments are available at www.plexdb.org [63] under accession numbers NF2 (wheat infection), NF3 (growth on complete medium) and FG18 (growth on putrescine medium).

## Supporting Information

Figure S1 **Alignment of four Pac2-like** ***Fusarium*** **orthologs.** Protein sequence alignment of four *Fusarium* Pac2-like proteins: Fo Pac2 (FOXG_12728) from *F. oxysporum*, FVEG_11476 from *F. verticillioides*, Fg Fgp2 (FGSG_10796) from *F. graminearum* and Fs_60837 from *F. solani* (*Nectria haematococca*). Conserved and similar residues are shaded gray. The solid black line represents the WOPRa box and the dashed black line the WOPRb box. The protein alignment was created using MacVector version 10.6.0.
(TIF)

Figure S2 **Analysis of transformants deleted for** ***FGP1 and FGP2*** **in** ***F. graminearum*** **.** A knock-out construct containing a hygromycin resistance gene coupled with the fluorescence GFP gene was introduced in the wild type strain PH-1. A) Schematic representation of the knock-out strategy for *FGP1* drawn to scale. B) PCR analysis was performed to verify homologous recombination and the absence of wild type *FGP1* in deletion strains using primers F1, R1 and R2 (Table S10). The picture shows the specific PCR amplification product obtained with primer F1 and R1 in the wild type and the specific PCR amplification product obtained with primer F1 and R2 in a correct deletion strain. C) Southern analysis was performed to verify correct homologous recombination at the *FGP1* locus in the *FGP1* deletion mutants. To this end, chromosomal DNA of wild type and the various mutants was digested with *Bsp*HI, blotted and hybridized with a probe corresponding to the *FGP1* downstream region. The *FRP1* locus of the wild type strain (WT) is visible as a 9.1 kb fragment. In the five *FGP1* deletion mutants, introduction of the gene replacement cassette by homologous recombination led to the expected replacement of the 9.1 kb fragment by a fragment of 7.4 kb. D) Schematic representation of the knock-out strategy for *FGP2* drawn to scale. E) PCR analysis was performed to verify homologous recombination and the absence of wild type *FGP2* in deletion strains using primers F2, R3 and R2 (Table S10). The picture shows the specific PCR amplification product obtained with primer F2 and R3 in the wild type and the specific PCR amplification product obtained with primer F2 and R2 in a correct deletion strain F) Southern analysis was performed to verify correct homologous recombination at the *FGP2* locus in the *FGP2* deletion mutants. To this end, chromosomal DNA of wild type and the various mutants was digested with *Bgl*II and *Sal*I, blotted and hybridized with a probe corresponding to the *FGP2* upstream region. The *FRP2* locus of the wild type strain (WT) is visible as a 8.7 kb fragment. In the five *FGP2* deletion mutants introduction of the gene replacement cassette by homologous recombination led to the expected replacement of the 8.7 kb fragment by a fragment of 2.8 kb.
(TIF)

Figure S3 **Comparison of GC-MS spectra, including trichothecene toxins, between wild type and the** ***FGP1*** **deletion strain.** No DON and 15ADON peaks are present in the GC-MS spectrum of the sample from a *FGP1* deletion strain grown in putrescine for 40 hours compared to the spectrum of the wild type sample.
(TIF)

Figure S4 **Formation of bulbous hyphae parallels the expression of** ***TRI*** **genes.** Time course of wild type (left) and a *FGP1* deletion strain (right) grown in putrescine medium at 8, 16, 24, 32, 40 and 48 HPI. Arrows indicate bulbous structures forming in putrescine medium.
(TIF)

Figure S5 **Differential gene expression** ***in vitro*** **and** ***in planta*** **.** Venn diagrams of genes expressed >2-fold higher in wild type PH-1 compared to the *FGP1* deletion strain during growth in putrescine medium, during wheat head infection or genes that are expressed exclusively during plant infection as reported by Lysøe *et al.* [26] (upper diagram) and of genes expressed >2-fold higher in the *FGP1* deletion strain compared to wild type PH-1 during growth in putrescine medium or during wheat head infection or genes that are expressed exclusively during plant infection (lower diagram).
(TIF)

Figure S6 **Comparative transcriptomics of Δ** ***fgp1*** **and Δ** ***sge1*** **mutants in** ***F. graminearum*** **and** ***F. oxysporum*** **, respectively.** Venn diagrams of orthologous genes expressed >2-fold higher in wild type PH-1 compared to the *FGP1* deletion strain of *F. graminearum* and genes expressed >2-fold higher in wild type *Fol*4287 compared to the *SGE1* deletion strain of *F. oxysporum* during growth in complete medium (upper diagram). Orthologous genes expressed >2-fold higher in the *FGP1* deletion strain compared to wild type PH-1 of *F. graminearum* and genes expressed >2-fold higher in the *SGE1* deletion strain compared to wild type *Fol*4287 of *F. oxysporum* during growth in complete medium (lower diagram).
(TIF)

Figure S7 **Quantification of expression levels for genes involved in conidiation.** Quantitative PCR shows that different conidiation genes are expressed at a lower level in the *SGE1* or the *FGP1* deletion strains compared to their respective wild type strain when grown in complete medium (CM), on mung bean agar (MBA) or in carboxymethylcellulose (CMC) medium. The relative expression levels of the different conidiation genes using the ΔCt method and *FRP1* as reference gene are represented in histograms. Three genes of *F. oxysporum*, *REN1* (FOXG_10430), *FLBC* (FOXG_01756) and *ABA1* (FOXG_00850) show lower expression levels in the *SGE1* deletion strain compared to wild type. The orthologous genes in *F. graminearum* show a different pattern. Expression of *REN1* (FGSG_02471) shows no significant difference between the *FGP1* deletion strain and wild type regardless of the growth medium. Expression of *FLBC* (FGSG_07052) shows, when grown on MBA, no significant difference between the *FGP1* deletion strain and wild type; when grown in CMC, a small increase in the *FGP1* deletion strain compared to wild type; and when grown in CM, a significant decrease in the *FGP1* deletion strain compared to wild type. Expression of *ABA1* (FGSG_11850/1) shows a significant difference between the *FGP1* deletion strain and wild type when grown on MBA or in CMC but not when grown in CM.
(TIF)

Table S1 (**A**) **Percentage of conservation of Pac2-like** ***Fusarium*** **orthologs.** Percentage similarity for Pac2-like orthologs (Fg: Fgp2 (FGSG_10796) from *F. graminearum*, Fv: FVEG_11476 from *F. verticillioides*, Fo: Pac2 (FOXG_12728) from *F. oxysporum* and Fs: Fs_60837 from *F. solani* (*Nectria haematococca*)). (**B**) **Overview of the glutamine residue percentage in the Wor1-like and Pac2-like** ***Fusarium*** **orthologs.** Wor1-like proteins: Fg: Fgp1 (FGSG_12164) from *F. graminearum*, Fv: FVEG_09150 from *F. verticillioides*, Fo: Sge1 (FOXG_10510) from *F. oxysporum* and Fs: Fs_81912 from *F. solani* (*Nectria haematococca*) and Pac2-like proteins (Fgp2 (FGSG_10796) from *F. graminearum*, Fv: FVEG_11476 from *F. verticillioides*, Fg: Fo: Pac2 (FOXG_12728) from *F. oxysporum* and Fs: Fs_60837 from *F. solani* (*Nectria haematococca*).
(DOCX)

Table S2 **Genes expressed >2-fold lower in putrescine medium for the Δ** ***fgp1*** **strain.**
(XLS)

Table S3 **Genes expressed >2-fold lower during wheat head infection for the Δ** ***fgp1*** **strain.**
(XLS)

Table S4 **Genes expressed exclusively in wheat or barley.**
(XLS)

Table S5 **Genes regulated by Tri6** ***in planta*** **and regulated by Fgp1 in putrescine medium or regulated by Fgp1** ***in planta*** **.**
(XLS)

Table S6 **Genes co-expressed with Tri5.**
(XLS)

Table S7 **Genes differentially regulated between** ***Fusarium graminearum*** **and** ***F. oxysporum*** **.**
(XLS)

Table S8 **Orthologous genes regulated by Fgp1 and Sge1.**
(XLS)

Table S9 **Genes differentially regulated by Fgp1 in different environments.**
(XLS)

Table S10 **Oligonucleotide primers sequences used in this study.**
(DOCX)
