# Supplementary figures and images for: The Wor1-like Protein Fgp1 Regulates Pathogenicity, Toxin Synthesis and Reproduction in the Phytopathogenic Fungus Fusarium graminearum
Source: PLoS Pathog. 2012 May 31;8(5):e1002724. doi: 10.1371/journal.ppat.1002724 (PMC3364952; doi:10.1371/journal.ppat.1002724)

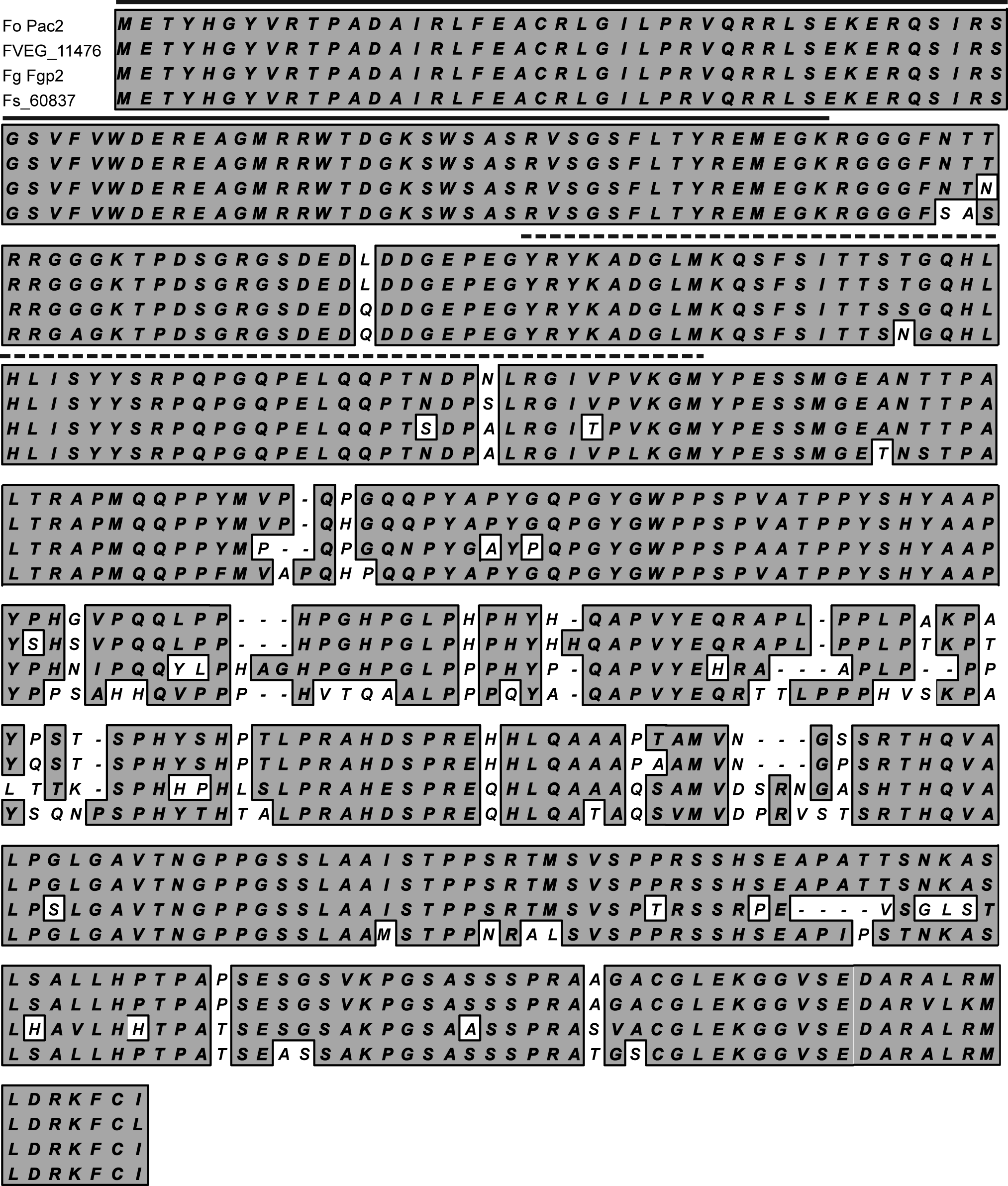

Supplement: Figure S1 — Alignment of four Pac2-like Fusarium orthologs. Protein sequence alignment of four Fusarium Pac2-like proteins: Fo Pac2 (FOXG_12728) from F. oxysporum, FVEG_11476 from F. verticillioides, Fg Fgp2 (FGSG_10796) from F. graminearum and Fs_60837 from F. solani (Nectria haematococca). Conserved and similar residues are shaded gray. The solid black line represents the WOPRa box and the dashed black line the WOPRb box. The protein alignment was created using MacVector version 10.6.0. (TIF) [file ppat.1002724.s001.tif]

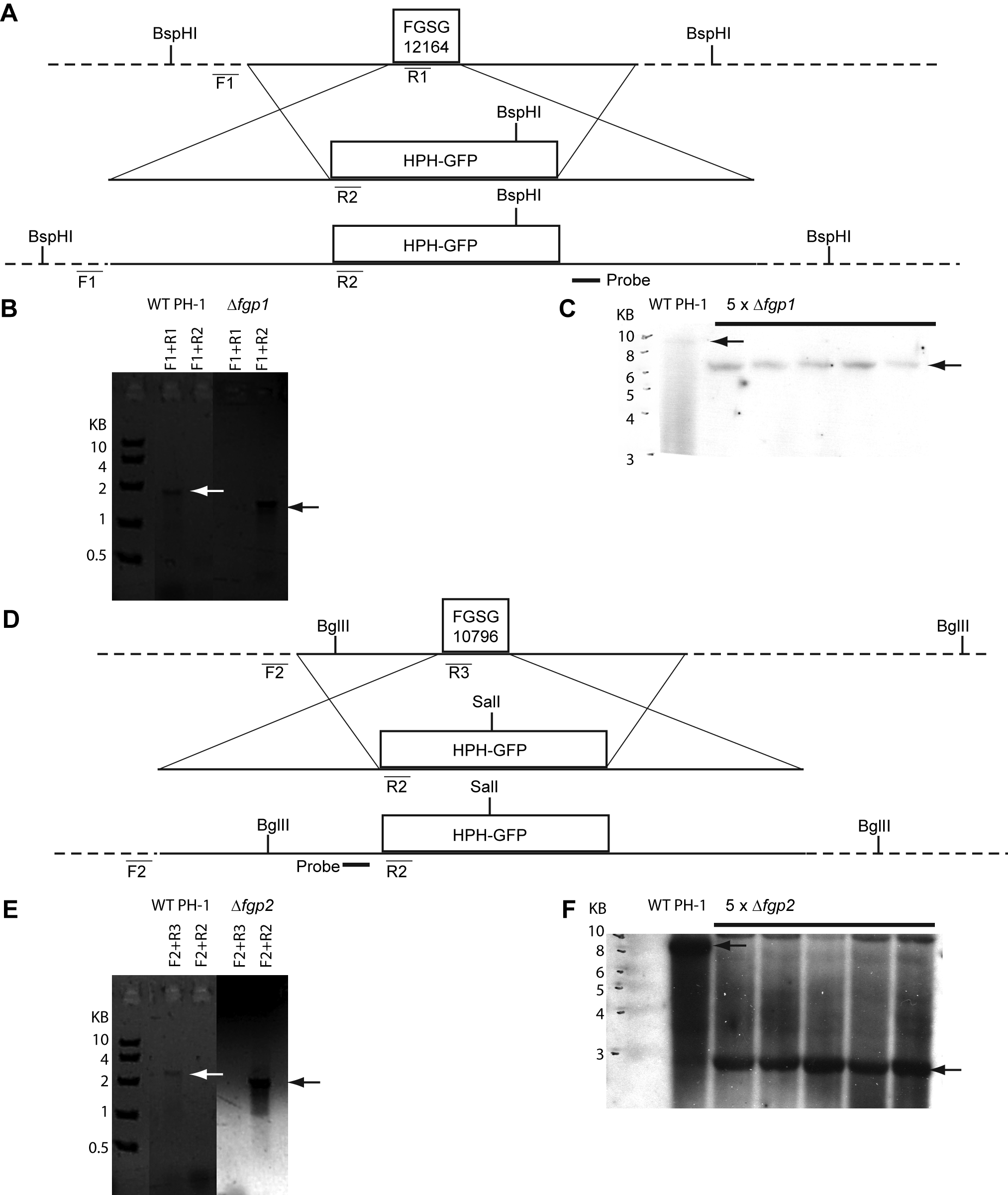

Supplement: Figure S2 — Analysis of transformants deleted for FGP1 and FGP2 in F. graminearum . A knock-out construct containing a hygromycin resistance gene coupled with the fluorescence GFP gene was introduced in the wild type strain PH-1. A) Schematic representation of the knock-out strategy for FGP1 drawn to scale. B) PCR analysis was performed to verify homologous recombination and the absence of wild type FGP1 in deletion strains using primers F1, R1 and R2 (Table S10). The picture shows the specific PCR amplification product obtained with primer F1 and R1 in the wild type and the specific PCR amplification product obtained with primer F1 and R2 in a correct deletion strain. C) Southern analysis was performed to verify correct homologous recombination at the FGP1 locus in the FGP1 deletion mutants. To this end, chromosomal DNA of wild type and the various mutants was digested with BspHI, blotted and hybridized with a probe corresponding to the FGP1 downstream region. The FRP1 locus of the wild type strain (WT) is visible as a 9.1 kb fragment. In the five FGP1 deletion mutants, introduction of the gene replacement cassette by homologous recombination led to the expected replacement of the 9.1 kb fragment by a fragment of 7.4 kb. D) Schematic representation of the knock-out strategy for FGP2 drawn to scale. E) PCR analysis was performed to verify homologous recombination and the absence of wild type FGP2 in deletion strains using primers F2, R3 and R2 (Table S10). The picture shows the specific PCR amplification product obtained with primer F2 and R3 in the wild type and the specific PCR amplification product obtained with primer F2 and R2 in a correct deletion strain F) Southern analysis was performed to verify correct homologous recombination at the FGP2 locus in the FGP2 deletion mutants. To this end, chromosomal DNA of wild type and the various mutants was digested with BglII and SalI, blotted and hybridized with a probe corresponding to the FGP2 upstream region. The FRP2 locus of [file ppat.1002724.s002.tif]

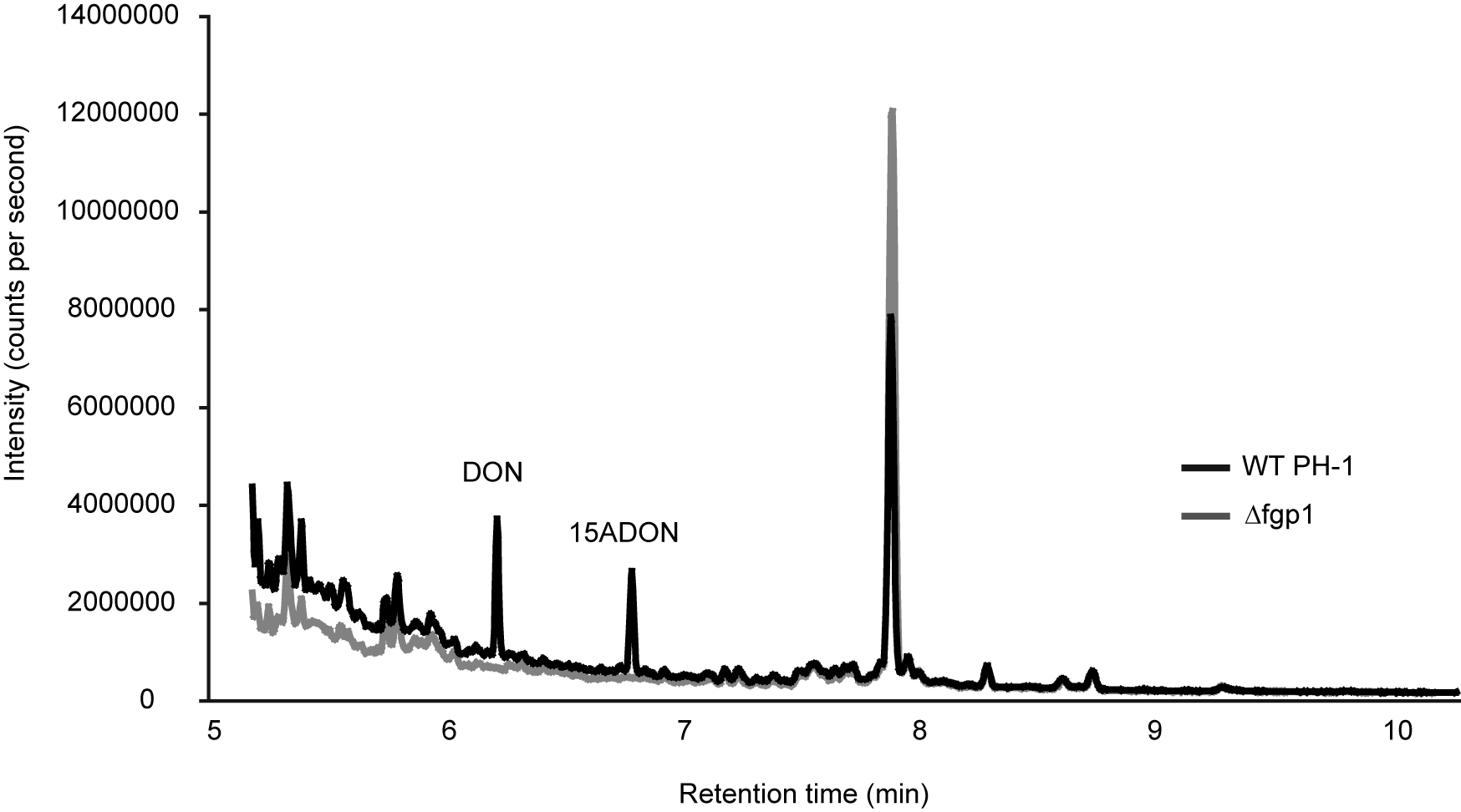

Supplement: Figure S3 — Comparison of GC-MS spectra, including trichothecene toxins, between wild type and the FGP1 deletion strain. No DON and 15ADON peaks are present in the GC-MS spectrum of the sample from a FGP1 deletion strain grown in putrescine for 40 hours compared to the spectrum of the wild type sample. (TIF) [file ppat.1002724.s003.tif]

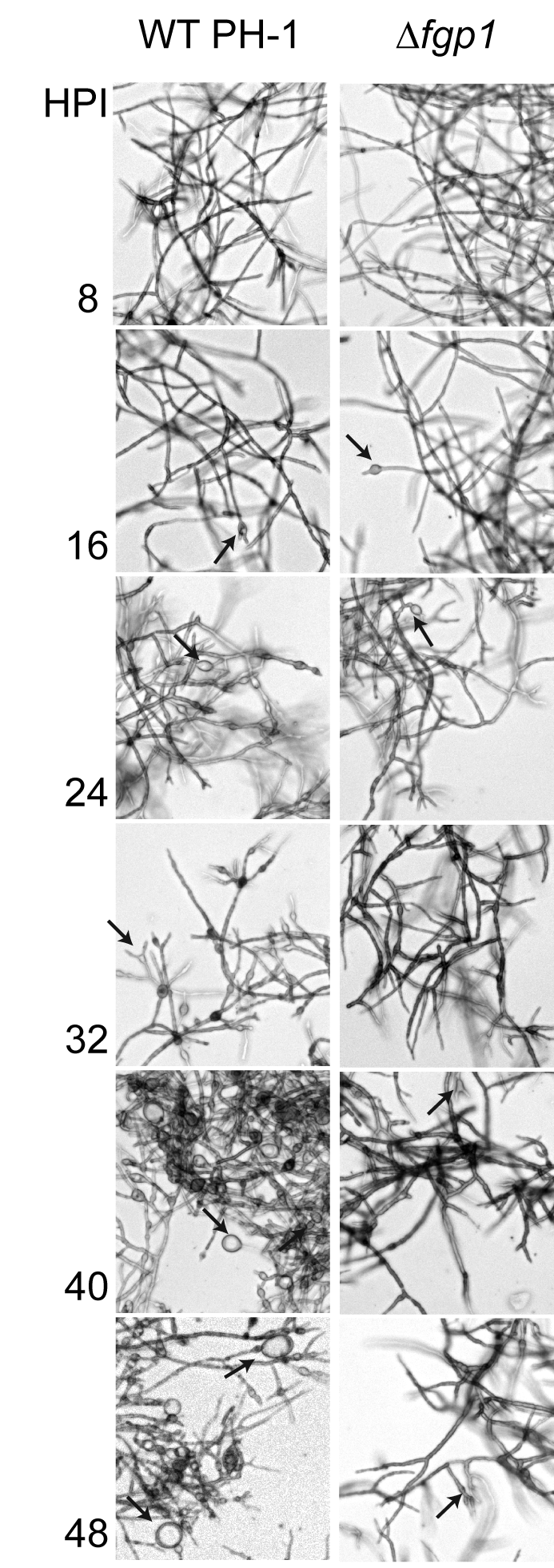

Supplement: Figure S4 — Formation of bulbous hyphae parallels the expression of TRI genes. Time course of wild type (left) and a FGP1 deletion strain (right) grown in putrescine medium at 8, 16, 24, 32, 40 and 48 HPI. Arrows indicate bulbous structures forming in putrescine medium. (TIF) [file ppat.1002724.s004.tif]

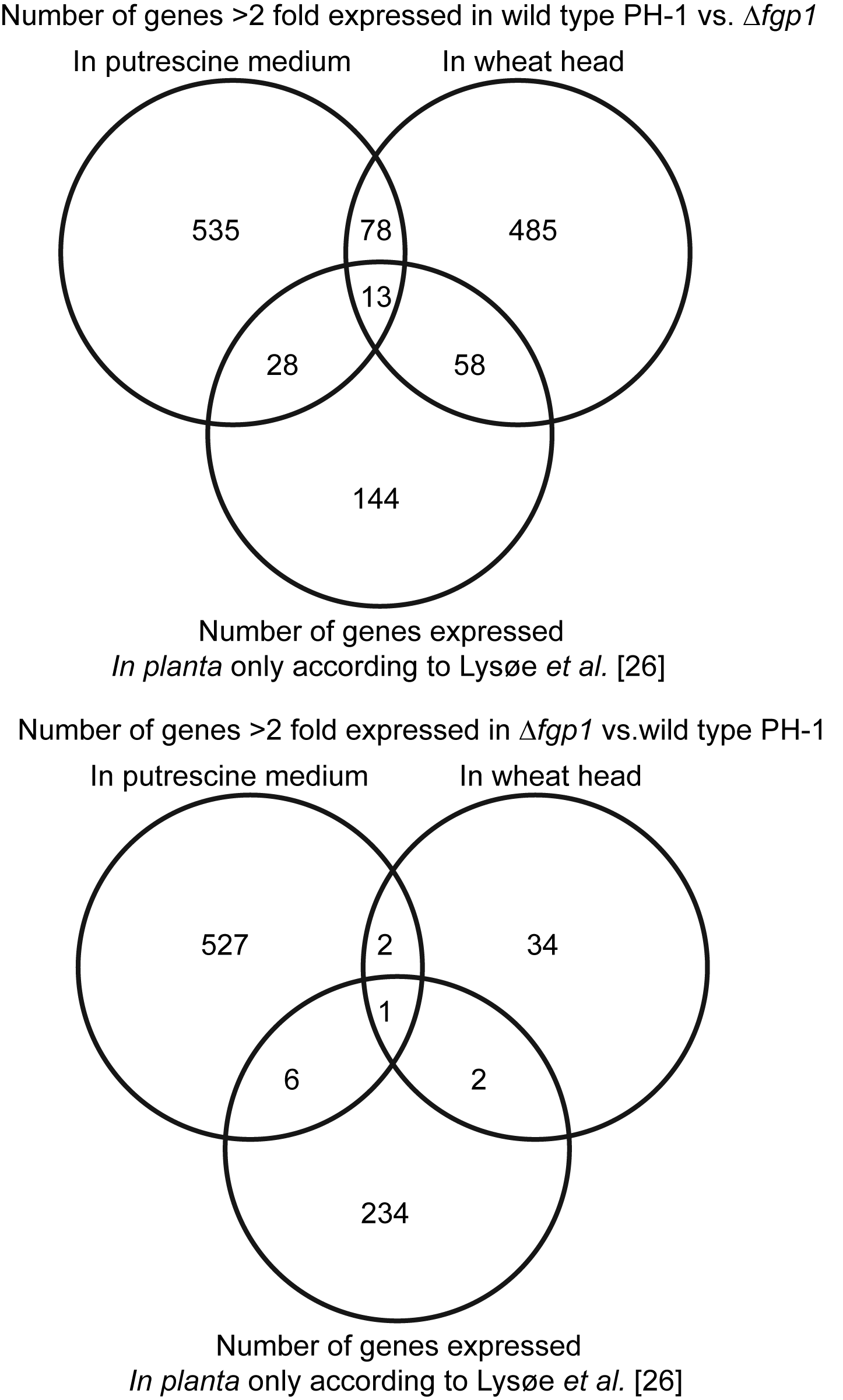

Supplement: Figure S5 — Differential gene expression in vitro and in planta . Venn diagrams of genes expressed >2-fold higher in wild type PH-1 compared to the FGP1 deletion strain during growth in putrescine medium, during wheat head infection or genes that are expressed exclusively during plant infection as reported by Lysøe et al. [26] (upper diagram) and of genes expressed >2-fold higher in the FGP1 deletion strain compared to wild type PH-1 during growth in putrescine medium or during wheat head infection or genes that are expressed exclusively during plant infection (lower diagram). (TIF) [file ppat.1002724.s005.tif]

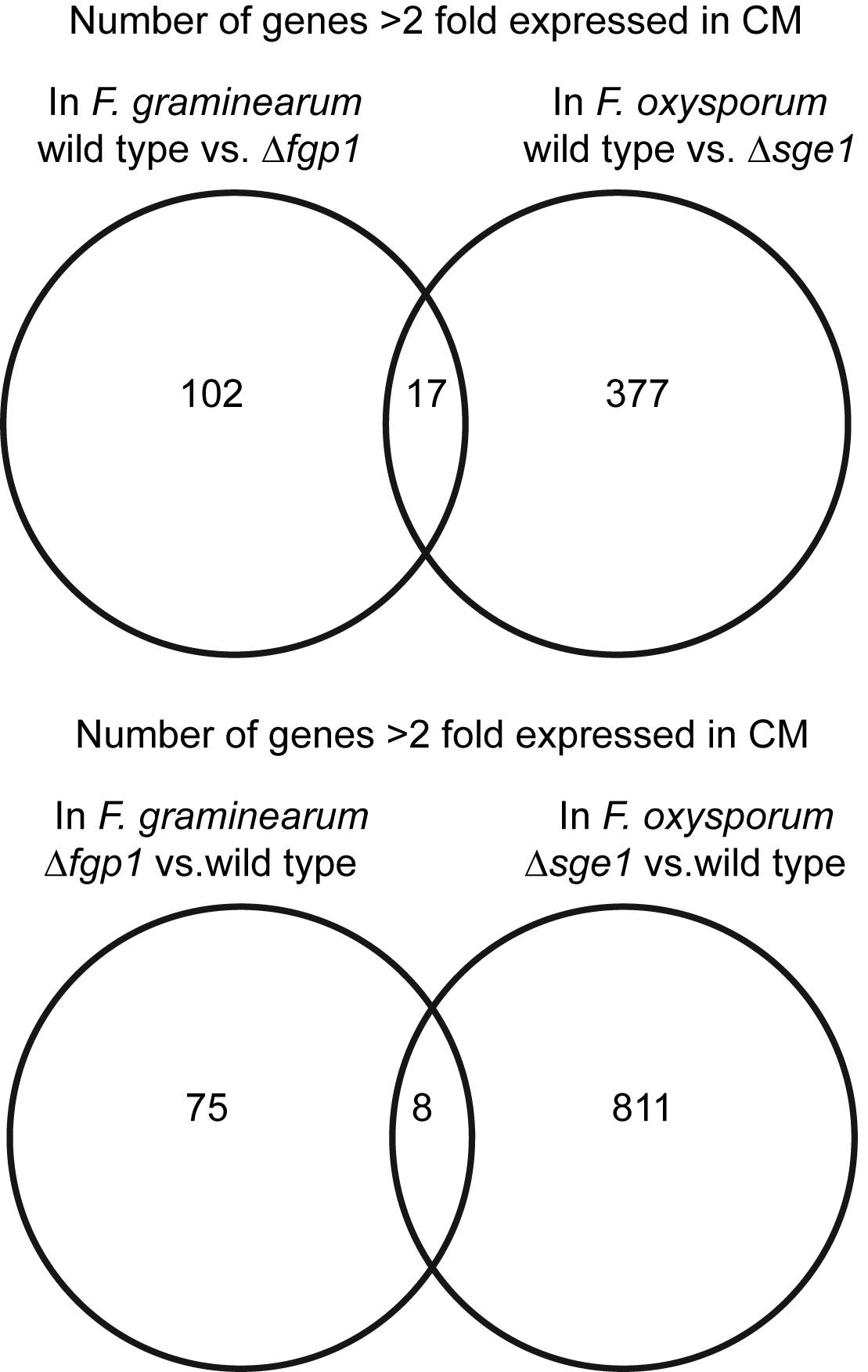

Supplement: Figure S6 — Comparative transcriptomics of Δ fgp1 and Δ sge1 mutants in F. graminearum and F. oxysporum , respectively. Venn diagrams of orthologous genes expressed >2-fold higher in wild type PH-1 compared to the FGP1 deletion strain of F. graminearum and genes expressed >2-fold higher in wild type Fol4287 compared to the SGE1 deletion strain of F. oxysporum during growth in complete medium (upper diagram). Orthologous genes expressed >2-fold higher in the FGP1 deletion strain compared to wild type PH-1 of F. graminearum and genes expressed >2-fold higher in the SGE1 deletion strain compared to wild type Fol4287 of F. oxysporum during growth in complete medium (lower diagram). (TIF) [file ppat.1002724.s006.tif]

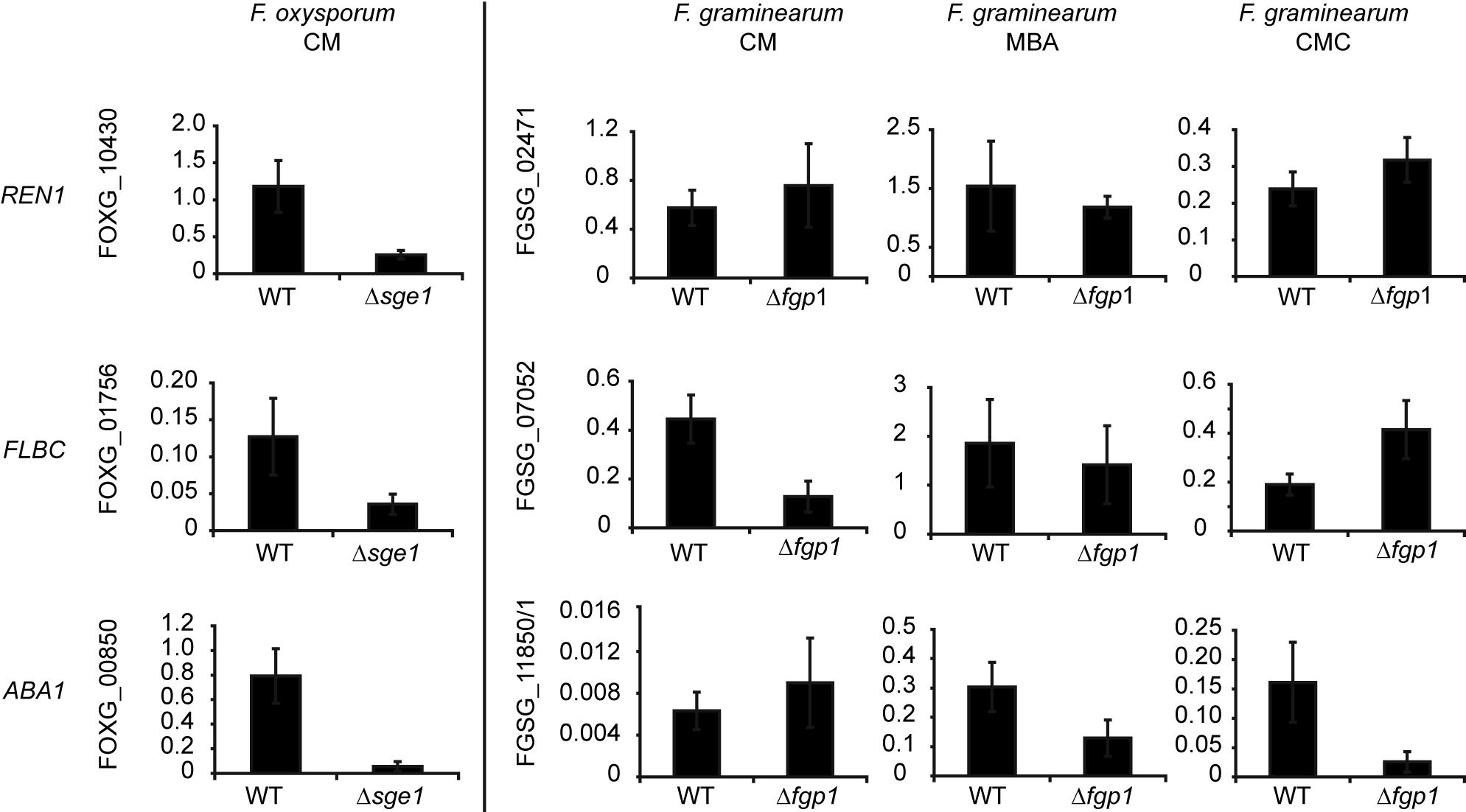

Supplement: Figure S7 — Quantification of expression levels for genes involved in conidiation. Quantitative PCR shows that different conidiation genes are expressed at a lower level in the SGE1 or the FGP1 deletion strains compared to their respective wild type strain when grown in complete medium (CM), on mung bean agar (MBA) or in carboxymethylcellulose (CMC) medium. The relative expression levels of the different conidiation genes using the ΔCt method and FRP1 as reference gene are represented in histograms. Three genes of F. oxysporum, REN1 (FOXG_10430), FLBC (FOXG_01756) and ABA1 (FOXG_00850) show lower expression levels in the SGE1 deletion strain compared to wild type. The orthologous genes in F. graminearum show a different pattern. Expression of REN1 (FGSG_02471) shows no significant difference between the FGP1 deletion strain and wild type regardless of the growth medium. Expression of FLBC (FGSG_07052) shows, when grown on MBA, no significant difference between the FGP1 deletion strain and wild type; when grown in CMC, a small increase in the FGP1 deletion strain compared to wild type; and when grown in CM, a significant decrease in the FGP1 deletion strain compared to wild type. Expression of ABA1 (FGSG_11850/1) shows a significant difference between the FGP1 deletion strain and wild type when grown on MBA or in CMC but not when grown in CM. (TIF) [file ppat.1002724.s007.tif]
