# Supplementary material for: The Wor1-like Protein Fgp1 Regulates Pathogenicity, Toxin Synthesis and Reproduction in the Phytopathogenic Fungus Fusarium graminearum
Source: PLoS Pathog. 2012 May 31;8(5):e1002724. doi: 10.1371/journal.ppat.1002724 (PMC3364952; doi:10.1371/journal.ppat.1002724)
Supplement: Table S1 — (A) Percentage of conservation of Pac2-like Fusarium orthologs. Percentage similarity for Pac2-like orthologs (Fg: Fgp2 (FGSG_10796) from F. graminearum, Fv: FVEG_11476 from F. verticillioides, Fo: Pac2 (FOXG_12728) from F. oxysporum and Fs: Fs_60837 from F. solani (Nectria haematococca)). (B) Overview of the glutamine residue percentage in the Wor1-like and Pac2-like Fusarium orthologs. Wor1-like proteins: Fg: Fgp1 (FGSG_12164) from F. graminearum, Fv: FVEG_09150 from F. verticillioides, Fo: Sge1 (FOXG_10510) from F. oxysporum and Fs: Fs_81912 from F. solani (Nectria haematococca) and Pac2-like proteins (Fgp2 (FGSG_10796) from F. graminearum, Fv: FVEG_11476 from F. verticillioides, Fg: Fo: Pac2 (FOXG_12728) from F. oxysporum and Fs: Fs_60837 from F. solani (Nectria haematococca). (DOCX) [file ppat.1002724.s008.docx]

**Table S1a: Percentage of conservation of Pac2-like *Fusarium* orthologs.**

Table in which the percentages of similarities are given for each of the Pac2-like ortholog (Fg: Fgp2 (FGSG_10796) from *F. graminearum,* Fv: FVEG_11476 from *F. verticillioides,* Fo: Pac2 (FOXG_12728) from *F. oxysporum* and Fs: Fs_60837 from *F. solani* (*Nectria haematococca*)) to each of the other protein.

|  | Fg | Fv | Fo | Fs |
| --- | --- | --- | --- | --- |
| Fg | - |  |  |  |
| Fv | 83 | - |  |  |
| Fo | 85 | 97 | - |  |
| Fs | 80 | 83 | 84 | - |

**Table S1b: Overview of the glutamine residue percentage in the Wor1-like and Pac2-like *Fusarium* orthologs.**

Wor1-like proteins: Fg: Fgp1 (FGSG_12164) from *F. graminearum*, Fv: FVEG_09150 from *F. verticillioides,* Fo: Sge1 (FOXG_10510) from *F. oxysporum* and Fs: Fs_81912 from *F. solani* (*Nectria haematococca*) and Pac2-like proteins (Fgp2 (FGSG_10796) from *F. graminearum,* Fv: FVEG_11476 from *F. verticillioides,* Fg: Fo: Pac2 (FOXG_12728) from *F. oxysporum* and Fs: Fs_60837 from *F. solani* (*Nectria haematococca*).

|  | # gln (%) in Wor-like orthologs | # gln (%) in Pac2-like orthologs |
| --- | --- | --- |
| Fg | 19 (5.6) | 21 (5.0) |
| Fv | 35 (10.7) | 21 (4.9) |
| Fo | 45 (13.6) | 20 (4.7) |
| Fs | 39 (11.5) | 25 (5.8) |
