# Supplementary material for: The Wor1-like Protein Fgp1 Regulates Pathogenicity, Toxin Synthesis and Reproduction in the Phytopathogenic Fungus Fusarium graminearum
Source: PLoS Pathog. 2012 May 31;8(5):e1002724. doi: 10.1371/journal.ppat.1002724 (PMC3364952; doi:10.1371/journal.ppat.1002724)
Supplement: Table S10 — Oligonucleotide primers sequences used in this study. (DOCX) [file ppat.1002724.s017.docx]

**Table S10**. Oligonucleotide primers sequences used in this study.

| 1 | Fg12164 F up locus (F1) | caaagcaaagcaacgcacag |
| --- | --- | --- |
| 2 | Fg12164 F up | tttttaattaacggtctcggtcccaa |
| 3 | Fg12164 R up | tttggtacctaagtgttgcgagtgttgcg |
| 4 | Fg12164 R locus (R1) | ATAGGTTGCTGTGAGCACTG |
| 5 | R up pPK2-hphgfp (R2) | caggtacacttgtttagaggt |
| 6 | Fg12164 F dwn | aaatctagagctagggtgaggggaaatt |
| 7 | Fg12164 R dwn | ccaggttcaagtccaagcttt |
| 8 | Fg10796 F up locus (F2) | cagaaccttaacgacttgatc |
| 9 | Fg10796 F up | aaattaattaacacggccccaagtgctt |
| 10 | Fg10796 R up | tttggtaCCATtgtgaaggtgtgtaacg |
| 11 | Fg10796 R up locus (R3) | AAGAGTCGAATAGCATCGGC |
| 12 | Fg10796 F dwn | aaatctagaaggagcagacagagcat |
| 13 | Fg10796 R dwn | tttaagcttaggtcaataacaggccgtgt |
| 14 | CompFg12164F1 | *ttttgaattc*tttgctgtcactcgtctgt |
| 15 | CompFg12164R1 | *ttttggta*cctgtctgtatgctaactag |
| 16 | F1SGE1BglII | ATGACCGTGAGCGTCAAGAT |
| 17 | R1SGE1Xba1 | aaaaTCtAgaGGGGCGCGGAAGTTCTGA |
| 18 | R2FGP1PmeIn | aaaaGTTTAAACGggtacctgtctgtatgcta |
| 19 | F2FGP1XbaI | tttttCTAGAGGATGGGGGGGAT |
| 20 | F3FGP1AhdI | ACCACCTCGTGAGCTACTAT |
| 21 | R3FGP1XbaI | aaaaTCTAGaGGGACCCTGAAGTTCTGTC |
| 22 | F4SGE1Xba1n | tttttcTaGAGGAAGCTCAACACGGaA |
| 23 | R4SGE1BglIIn | aaaaagatcttattgcaatcagcggcctt |
| 24 | Fg12164 Sf | gctagggtgaggggaaatt |
| 25 | Fg12164 Sr | ctgaatcgcatgggtcattg |
| 26 | Fg10796 Sf | ctgactagcgctgtcactat |
| 27 | Fg10796 Sr | Ccattgtgaaggtgtgtaacg |
| 28 | Tri14 coding F | ATTTTGAGCCATCTCGGTTC |
| 29 | Tri14 coding R | TTAGATCTCGTCGCGGACCT |
| 30 | Actin F | TCAACCCCAAGTCCAACC |
| 31 | Actin R | AGGCGTACAGGGACAGAA |
| 30 | FoFrp1Fq | ATGGATCCGCCACATATCACCGAA |
| 31 | FoFrp1Rq | ACGCTGATGTTGGTGTGCATTGAG |
| 32 | FgFrp1Fq | TTATGTGGCCTTTGGCACCGATTG |
| 33 | FgFrp1Rq | AAAGCAATTCGCTGGGATTCGGTC |
| 34 | FoAba1Fq | AGACCCAGAAATGGTGTCAACCCT |
| 35 | FoAba1Rq | ATGGAGCTTGCCACCCATAGAGAA |
| 36 | FgAba1Fq | AAAGACACCCAGAAGGACAAGGGT |
| 37 | FgAba1Rq | ATAAGCATGTTGCGCCCATGTAGC |
| 38 | FoRen1Fq | TACCTGCCAATCGGTAAGCGTCAA |
| 39 | FoRen1Rq | ACCGATGTGACATAACACTCGCCT |
| 40 | FgRen1Fq | ACGACAGACTTGAATCGCCTGACA |
| 41 | FgRen1Rq | TATCGTGCCACATCGTATCCAGCA |
| 42 | FoFlb1Fq | GCAGCACTTCTTCAATGGCCTCAT |
| 43 | FoFlb1Rq | AGGTGCAAATTGGTGGTTGACAGG |
| 44 | FgFlb1Fq | TTCAGCTCCAAGGTGTCTTCCAGT |
| 45 | FgFlb1Rq | ACAGAGAAATGTCGACCACAGCCT |
